# Orthobiologic therapies delay the need for hip arthroplasty in patients with avascular necrosis of the femoral head: A systematic review and survival analysis

**DOI:** 10.1002/ksa.12532

**Published:** 2024-11-14

**Authors:** Marco Zaffagnini, Angelo Boffa, Luca Andriolo, Federico Raggi, Stefano Zaffagnini, Giuseppe Filardo

**Affiliations:** ^1^ Clinica Ortopedica e Traumatologica 2, IRCCS Istituto Ortopedico Rizzoli Bologna Italy; ^2^ Applied and Translational Research (ATR) Center, IRCCS Istituto Ortopedico Rizzoli Bologna Italy; ^3^ Department of Surgery EOC, Service of Orthopaedics and Traumatology Lugano Switzerland; ^4^ Faculty of Biomedical Sciences Università Della Svizzera Italiana Lugano Switzerland

**Keywords:** avascular necrosis, hip, orthobiologics, osteonecrosis, regenerative therapies

## Abstract

**Purpose:**

The aim of this systematic review and survival analysis was to quantify the benefits of orthobiologic augmentation therapies for the treatment of avascular necrosis (AVN) of the femoral head and identify the most effective approach to delay the need for total hip arthroplasty (THA).

**Methods:**

A systematic review of the literature was performed on PubMed, Scopus, and Cochrane on clinical studies on orthobiologic therapies used alone or as an augmentation to core decompression or other procedures to address hip AVN. A qualitative analysis of the different biological therapies applied was performed. Afterward, the results of these procedures were quantitatively analysed to document their survivorship from THA compared to treatment groups without orthobiologics. Kaplan–Meier analysis was performed for all studies and then by categorising orthobiologics into treatment subgroups.

**Results:**

A total of 106 studies were included (4505 patients). Different orthobiologic approaches have been evaluated: cell‐based therapies including bone marrow aspirate concentrate (BMAC) and bone marrow mesenchymal stromal cells (BM‐MSCs), platelet‐rich plasma (PRP), or other bioactive molecules applied in the osteonecrotic area or as intra‐arterial injections. The survival analysis at 120 months documented a higher (*p* < 0.0005) cumulative survivorship with orthobiologics (69.4%) compared to controls (48.5%). The superiority was shown specifically for BMAC (*p* < 0.0005), BM‐MSCs (*p* < 0.0005), intra‐arterial (*p* < 0.0005) and PRP (*p* = 0.011) approaches, but the direct comparison of these approaches with their controls confirmed benefits only for BMAC (*p* < 0.0005).

**Conclusion:**

This systematic review and survival analysis demonstrated that orthobiologics have the potential to improve survivorship in patients affected by hip AVN. In particular, the specific analysis of different orthobiologic products supported relevant benefits for BMAC augmentation in terms of survival from the need for THA, while no clear benefits were confirmed for other orthobiologics.

**Level of Evidence:**

Level III.

**Abbreviations**
AVN: avascular necrosis
BMAC: bone marrow aspirate concentrate
BM‐MSCs: bone marrow mesenchymal stromal cells
CD: core decompression
ESWT: extracorporeal shock wave therapy
G‐CSF: granulocyte‐colony stimulating factor
hUC‐MSCs: human umbilical cord‐derived mesenchymal stromal cells
MSCs: mesenchymal stromal cells
PRP: platelet‐rich plasma
RCTs: randomised controlled trials
rhBMP: recombinant human bone morphogenetic protein
THA: total hip arthroplasty

## INTRODUCTION

Osteonecrosis or avascular necrosis (AVN) of the femoral head is one of the most frequent hip‐disabling pathologies commonly affecting young people in the third and fourth decades of life [80, 88]. Total hip arthroplasty (THA) is an increasingly common surgical procedure in the United States, reaching more than 300,000 procedures per year, of which up to 18% is due to AVN [53, 97]. When performed after AVN, THA is associated with worse outcomes compared to joint replacement in primary hip osteoarthritis, with higher periprosthetic fractures, infections and revision rates [97, 104]. Moreover, patients affected by AVN are young and active, and thus revision surgeries may be required more frequently in these patients' lifetimes [97]. As a result, there is a growing interest in the investigation of early interventions for AVN aimed at preserving the native hip to avoid or at least delay the need for THA [40].

Core decompression (CD) represents the first‐line treatment for patients with low AVN grades to reduce intraosseous pressure and promote the repair processes [3, 40, 58]. Other approaches have been proposed over the years including bone substitutes, scaffolds or metal constructs applied to reinforce the bone architecture and reduce the risks of femoral head collapse [28, 131]. However, despite some promising results, the survival rate from THA of these procedures is not satisfactory; consequently, no treatment has been universally accepted as a gold standard [45, 80]. Thus, the augmentation of orthobiologics to these procedures has been introduced in the clinical practice to exploit the biological potential of mesenchymal stromal cells (MSCs), growth factors and bioactive molecules with the aim to delay the collapse of the femoral head and consequently the need for THA [51]. Among these, cell‐based therapies are a common approach applied in AVN management and cell sources are numerous. The most used is bone marrow due to its relative accessibility, availability and the potential for point‐of‐care use [24]. Otherwise, MSCs can be harvested from the abdomen or the human umbilical cord [46, 123, 125]. Followed by cells, platelet‐rich plasma (PRP) has been widely studied over time, alone or associated with other biological treatments, for the treatment of hip AVN with the aim to stimulate bone formation and vascularisation, as well as to counteract adipogenesis [119]. Additionally, recombinant human bone morphogenetic protein (rhBMP) is largely used for its properties in enhancing osteogenesis of human MSCs, depositing bone extracellular matrix and achieving a mature osteoblast phenotype [100]. Finally, intra‐arterial orthobiologic treatments have been proposed for their role in improving oxidative stress parameters and decreasing the expression and inflammasomes [113]. All these orthobiologic options showed a biologic rationale and promising results, but indications on the most suitable approach are needed. Although orthobiologic therapies seem to improve the outcomes of these procedures [3], it is still unclear which orthobiologic treatment is the most effective in delaying the need for prostheses in patients with AVN.

The aim of this systematic review and survival analysis was to quantify the benefits of orthobiologic augmentation therapies for the treatment of AVN and identify the most effective approach to delay the need for THA.

## MATERIALS AND METHODS

A systematic review of the literature was performed on orthobiologic therapies for AVN treatment. The search was conducted on 15 February 2024 on three medical electronic databases (PubMed, Scopus and Cochrane), using the following parameters: ((Stem cells) OR (bone marrow) OR (mesenchymal bone marrow) OR (biological therapies) OR (regenerative therapies)) AND ((femoral head surgery) OR (femoral head decompression) OR (hip decompression)) AND ((osteonecrosis) OR (necrosis) OR (bone marrow oedema) OR (bone marrow pathology)). The guidelines for preferred reporting items for systematic reviews and meta‐analysis were used [79]. The screening process and analysis were conducted separately by two independent observers (M. Z. and A. B.). First, the articles were screened by title and abstract. The following inclusion criteria for relevant articles were used during this initial screening: clinical studies of any level of evidence, written in the English language, with no time limitation, on biological regenerative therapies used to treat patients affected by hip AVN. Exclusion criteria were articles written in other languages, preclinical studies, reviews or clinical studies on hip AVN analysing nonorthobiologic approaches. In the second step, the full texts of the selected articles were screened, with further exclusions according to the previously described criteria. Furthermore, articles that did not report clinical outcome data were excluded. Reference lists from the selected papers were also screened. Relevant data (type of study, type of treatment, number of patients, age of the patients, disease stage, aetiology, follow‐up, results, complications and failures) were extracted. All data were then collected in a unique database with the consensus of the two observers (M. Z. and A. B.) and analysed for the purposes of the present manuscript. In case of disagreement between the two authors, divergences were discussed with a third author (L. A.) and a consensus was reached.

The primary step of this study was to document, through qualitative analysis, the different types of biological therapies that are applied in clinical practice to treat hip AVN. Afterward, the results of these procedures were quantitatively analysed to document their clinical potential in terms of survivorship from THA compared to treatment groups without orthobiologics. Only studies reporting failure as the need for a THA procedure were included in the survival analysis. Studies were analysed with a Kaplan–Meier analysis if they reported, for every single failure, either the specific time of revision to THA or at least a failure time in an interval no longer than 2 years [23]. To this purpose, the number and timing of failures in terms of the need for THA have been derived from the text, while the ‘WebPlotDigitizer’ programme was used for studies only reporting Kaplan–Meier curve graphs [17]. Moreover, a further Kaplan–Meier analysis including only comparative studies was conducted. In addition, a Kaplan–Meier analysis was performed by categorising orthobiologics therapies into subgroups based on the treatment type, each one containing at least 100 patients, aiming to compare different approaches.

Finally, to perform a Level II analysis to verify the most effective orthobiologic approach, only comparative studies were considered. A separate Kaplan–Meier analysis was conducted for each orthobiologic subgroup with at least 100 patients including cases and controls.

## RESULTS

### Article selection

**Figure 1:**
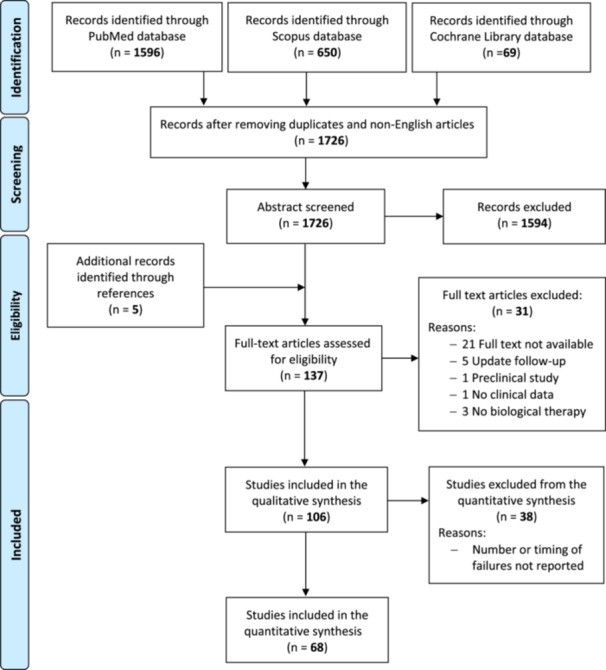
Preferred reporting items for systematic reviews and meta‐analyses flowchart of the study selection process.

**Figure 2:**
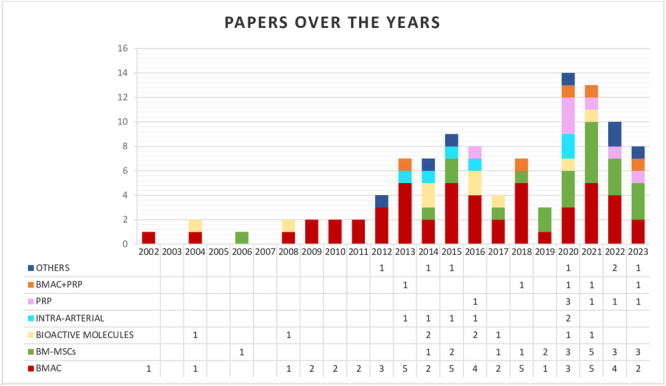
Number of articles published over time on the treatment of avascular necrosis of the femoral head with orthobiologic therapies. BMAC, bone marrow aspirate concentrate; BM‐MSCs, bone marrow mesenchymal stromal cells; PRP, platelet‐rich plasma.

The database search identified 1726 records, and the abstracts were screened and selected according to the inclusion/exclusion criteria. As shown in Figure 1, 137 full‐text articles were assessed for eligibility. A total of 31 articles did not fulfil the criteria and were further excluded, leading to a total of 106 studies included in the qualitative analysis: The detailed description of these studies is reported in the supplementary materials. As shown in Figure 2, almost half of the included studies were published in the last 5 years, showing an increment in the interest in these orthobiologic approaches. Overall, the evaluation of study type showed 19 randomised controlled trials (RCTs), five prospective comparative studies, 19 retrospective comparative studies, 60 case series and three case reports.

### Studies' characteristics

These studies reported the results of AVN orthobiologic treatment in 5652 hips of 4505 patients. The populations included in the selected studies were very heterogeneous in terms of age, aetiology and AVN stage. Age presented a wide range from children and teenagers to seniors (details in Supporting Information). Only six studies analysed specifically young populations, either affected by sickle cell disease [6, 82, 95], leukaemia [93], AVN after femoral neck fractures [27] or administration of steroid or chemotherapy [20]. Aetiology included steroid therapy (2072 hips), alcohol (931 hips), idiopathic (685 hips), sickle cell disease (559 hips), trauma (182 hips), multiple causes (39 hips), smoke (34 hips), immunosuppressive therapy (29 hips), lupus (24 hips), chemotherapy (15 hips), HIV (12 hips), Caisson disease (12 hips), hormonal (six hips), hepatitis C (three hips), Cushing disease (two hips), pregnancy (one hip), thalassaemia (one hip), octreotide (one hip), osteochondrosis dissecans (one hip), hypothyroidism (one hip), nontraumatic not specified aetiologies (46 hips) or other not specified aetiologies (253 hips).

AVN stage was evaluated with different systems: Association Research Circulation Osseous classification in 46 articles, Ficat classification in 30, Steinberg classification in 14, Japanese International Committee classification in 10, Japanese Orthopaedic Association classification in five and Mitchell classification in one study. Overall, lesions of different stages, from early‐stage AVN to collapsed head, were treated. Finally, evaluation time was also heterogeneous, with 24 studies reporting short‐term ( <24 months follow‐up), 59 medium‐term (24–60 months) and 21 long‐term results ( >60 months), while two studies did not specify the follow‐up. Further details are reported in the Supplementary materials (Supporting Information S1: Table S1).

### Qualitative analysis: Orthobiologics approaches

Different orthobiologic approaches and different surgical procedures have been evaluated in the included studies. More details on the specific treatments used in each study are reported in the supplementary materials. From a biological point of view, five different macro‐groups of treatment could be distinguished:

1. MSCs applied in the osteonecrotic area: 80 studies on bone marrow mesenchymal stromal cells (BM‐MSCs), applied as bone marrow aspirate concentrate (BMAC) in 50 studies [1, 6, 8, 14, 16, 17, 19, 21, 25, 26, 29, 35, 37, 38, 39, 41, 42, 43, 44, 47, 49, 54, 55, 57, 61, 62, 65, 66, 70, 78, 82, 86, 87, 89, 91, 101, 105, 107, 108, 109, 110, 111, 114, 115, 120, 121, 122, 126, 127, 131], 22 as cultured‐expanded [4, 5, 7, 20, 22, 30, 31, 32, 50, 56, 69, 74, 84, 93, 94, 95, 112, 117, 125, 130, 131, 132], three as bone marrow aspirates [10, 13, 76], two studies on adipose‐tissue‐derived MSCs [46, 129], two studies on allogeneic human umbilical cord‐derived MSCs (hUC‐MSCs) [28, 123] and one study on both BMAC and osteoblastic cell implantation [36].
2. MSCs applied as intra‐arterial injection in the collateral branches of the femoral artery, whole obturator artery and medial and lateral circumflex arteries: The types of cells used with this approach were peripheral blood MSCs mobilised by the granulocyte‐colony stimulating factor (G‐CSF) in three studies [73, 85, 124] and allogeneic hUC‐MSCs [11], bone marrow‐derived MSCs [72] and the combination of the last two methods [9] in one study each.
3. Bioactive molecules applied in the osteonecrotic area: Seven studies on the rhBMP, applied as rhBMP‐2 in three [27, 103, 106], three on rhBMP‐7 [10, 76, 102], one on partially purified human BMP [64] and two studies on the recombinant human fibroblast growth factor‐2 [59, 60].
4. PRP: Six studies evaluated PRP applied in the osteonecrotic area, with three studies on the association with CD [2, 33, 71] and three studies on the association with CD and bone graft [68, 98, 118]. One study evaluated PRP use through intra‐articular injection [67].
5. Combined orthobiologic therapies applied in the osteonecrotic area: Five studies on PRP + BMAC [18, 48, 63, 75, 92] and one study on PRP + adipose‐tissue derived MSCs + hyaluronic acid [83].

Regarding the treatments evaluated, with or without orthobiologic augmentation, most of the included studies focused CD procedure (90 studies), which was combined with synthetic bone grafts or scaffolds in 14 studies [10, 14, 18, 32, 38, 46, 47, 56, 62, 66, 68, 105, 120, 121], autologous bone graft in 11 studies [28, 54, 61, 65, 70, 98, 102, 103, 106, 115, 118], vascularised bone grafts in four studies [4, 5, 32, 132]), low‐intensity pulsed ultrasound in two studies [78, 105], bisphosphonates in two studies [29, 69] and with allografts [1], xenografts [10] and intravenous iloprost [89] in one study each. The other studies evaluated other approaches like metal constructs [28, 73, 124, 132], extracorporeal shock wave therapy (ESWT) [67], saline injections [35] or THA [39], while three studies compared orthobiologic therapies with no treatment [60, 109, 111].

### Clinical results

The clinical results of each study are reported in detail in the Supporting Information (Supporting Information S1: Table S1). In summary, most of the studies analysed in this systematic review showed clinical score improvements and good radiological and histological outcomes with orthobiologic therapies.

Among the 19 RCTs analysed, 15 studies confirmed overall favourable results for the biological augmentation, while only four studies did not find any significant difference. The 15 RCTs reporting a higher improvement of outcomes in the orthobiologic groups analysed BMAC in eight studies (out of 11 studies), PRP in three studies, BM‐MCSs and intra‐arterial treatments in two studies.

In detail, regarding the studies analysing BMAC, five RCTs evaluating BMAC reported better clinical outcomes for CD + BMAC treatment versus CD alone in two studies [101, 107], for CD + BMAC+autologous bone graft versus CD+autologous bone graft in two studies [61, 70] and for CD + BMAC + synthetic bone graft versus CD + synthetic bone graft in one study [62]. Besides the clinical improvement, Gangji et al. showed radiologic improvements for CD + BMAC treatment with respect to CD alone [25]. In addition, Hernigou et al. suggested the use of computer‐assisted surgery with respect to fluoroscopy to reduce radiation exposures and obtain a better repair of AVN in patients treated with CD + BMAC [44]. Moreover, Hauzeur et al. compared two orthobiologic approaches and did not find any superiority of osteoblastic cells with respect to BMAC [36].

Regarding the three RCTs evaluating PRP augmentation, better clinical outcomes were reported in favour of CD + PRP treatment versus CD alone in one article [2] and in favour of PRP versus ESWT in another study [67], while in the last study, both clinical and radiological superiorities were reported for CD + PRP + autologous grafts approach versus CD+autologous grafts [118].

Regarding the two RCTs evaluating BM‐MCSs, one study reported better clinical outcomes for the CD + BM‐MSC approach versus CD alone [131] and one study documented both clinical and radiological improvements of hips treated with CD + BM‐MSCs compared to CD alone [130].

Finally, two RCTs analysed intra‐arterial treatments: Zhang et al. demonstrated superior vascularisation capacity, both autonomously and through paracrine mechanisms, in the intra‐arterial autologous lipoaspirate cell approach compared to saline [129], and Mao et al. reported better clinical outcomes for intra‐arterial peripheral blood MSCs mobilised by G‐CSF + biomechanical support approach versus biomechanical support alone [73].

On the other hand, four RCTs did not demonstrate the superiority of orthobiologic augmentation compared to the control group. In detail, Pepke et al. [15] reported comparable clinical scores and failure rates, analysing at 2‐year follow‐up 11 hips treated with CD and BMAC implantation against 14 hips treated with CD alone. Moreover, Rastogi et al. [23] compared 30 hips treated with CD and unprocessed bone marrow with 30 hips treated with CD and BMAC implantation, finding a significant difference in radiological scores, but none in clinical scores. In addition, Hauzeur et al. did not find any improvement in the evolution of AVN stage 3 with the implantation of BMAC after CD [35]. Finally, Jayankura et al. did not show any advantage in the use of autologous osteoblastic cells versus saline to improve the results of CD in patients with early‐stage AVN [50].

### Survival analysis: Orthobiologic versus nonorthobiologic approaches

**Figure 3:**
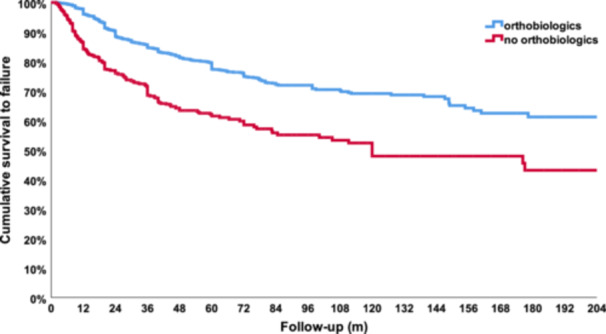
Comparison between the survivorship curves of hips treated with orthobiologic therapies (blue line) versus control groups (red line). m, months.

The survival analysis of failures evaluated 3322 hips analysed in 68 studies (excluding studies not reporting the number or the time of failures). The Kaplan–Meier analysis of 2530 hips treated with orthobiologic therapies showed a total estimated cumulative survivorship of 96.1% at 12 months of follow‐up, 77.3% at 60 months of follow‐up, 69.4% at 120 months of follow‐up and 62.9% at 180 months of follow‐up (Figure 3). Conversely, the hips treated without orthobiologic treatments that are part of the control groups in the comparative studies were analysed in a different Kaplan–Meier analysis. This Kaplan–Meier analysis of 792 hips showed a total estimated cumulative survivorship of 84.3% at 12 months of follow‐up, 61.8% at 60 months of follow‐up, 48.5% at 120 months of follow‐up and 43.5% at 180 months of follow‐up (Figure 3). The analysis of the two curves showed a statistically significant difference (*p* < 0.0005) confirming overall superior results and durability in favour of orthobiologic therapies compared to control groups.

**Figure 4:**
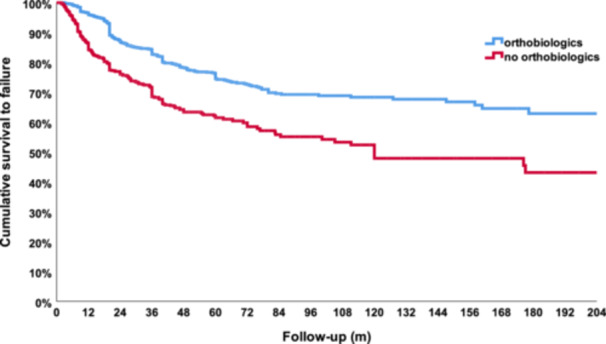
Comparison between the survivorship curves of hips of comparative studies treated with orthobiologic therapies (blue line) versus control groups (red line). m, months.

The Kaplan–Meier analysis including only comparative studies was performed on 23 studies for a total of 1847 hips analysed. The total estimated cumulative survivorship for orthobiologic therapies and control groups was 95.5% versus 86.8% at 12 months of follow‐up, 75.5% versus 62.1% at 60 months of follow‐up, 68.1% versus 48.1% at 120 months of follow‐up and 63.0% versus 43.2% at 180 months of follow‐up, with a statistically significant difference between the two curves (*p* < 0.0005), showing the benefit of orthobiologic augmentation therapies to provide longer lasting results for the treatment of AVN (Figure 4).

### Survival analysis on different orthobiologic approaches

**Figure 5:**
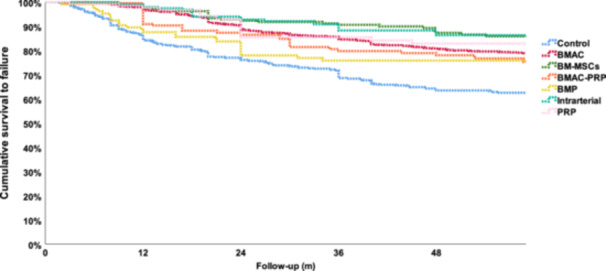
Comparison between the hip survivorship curves of studies treated with different orthobiologic therapies. BMAC, bone marrow aspirate concentrate; BMP, bone morphogenetic protein; BM‐MSCs, bone marrow mesenchymal stromal cells; m, months; PRP, platelet‐rich plasma.

The categorisation of the orthobiologic therapies into subgroups based on the treatment types led to six subgroups with at least 100 patients each: BMAC (1624 patients), BM‐MSCs (299 patients), intra‐arterial treatments (190 patients), BMAC + PRP (186 patients), PRP (127 patients) and BMP (104 patients). The Kaplan–Meier analysis showed a statistically significant difference with higher survivorship for the BMAC subgroup (*p* < 0.0005), BM‐MSCs subgroup (*p* < 0.0005), intra‐arterial treatment subgroup (*p* < 0.0005) and PRP subgroup (*p* = 0.011) with respect to the control groups (Figure 5). On the other hand, BMAC + PRP and BMP did not show better results with respect to the control subgroup (Figure 5).

The direct comparison of the orthobiologic subgroups showed better results for the BM‐MSC subgroup compared to BMAC + PRP (*p* < 0.0005), BMP (*p* = 0.002) and PRP (*p* = 0.001), as well as better results for intra‐arterial treatments compared to BMAC + PRP (*p* = 0.005), BMP (*p* = 0.004) and PRP (*p* = 0.004). Finally, the BMAC subgroup showed better results compared to BMAC + PRP (*p* < 0.0005) and BMP (*p* = 0.024).

### Level II survival analysis on different orthobiologic approaches in comparative studies

**Figure 6:**
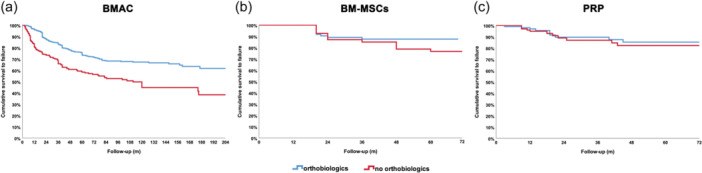
(a) Comparison between the survivorship curves of hips of comparative studies treated with BMAC (blue line) versus control groups (red line); (b) Comparison between the survivorship curves of hips of comparative studies treated with BM‐MSCs (blue line) versus control groups (red line); (c) Comparison between the survivorship curves of hips of comparative studies treated with PRP (blue line) versus control groups (red line). BMAC, bone marrow aspirate concentrate; BM‐MSCs, bone marrow mesenchymal stromal cells; m, months; PRP, platelet‐rich plasma.

Further analyses were performed including only comparative studies, analysing the differences in terms of survival between the orthobiologic group and the control group of each orthobiologic approach. The analysis was conducted only for orthobiologic approaches investigated in studies with a cumulative number of patients of at least 100: BMAC, BM‐MSCs and PRP. In the subgroup considering BMAC, a total of 1286 patients were analysed, of which 804 were treated with BMAC and 482 were without the orthobiologic treatment. The Kaplan–Meier analysis showed a statistically significant difference with higher survivorship for the BMAC subgroup (*p* < 0.0005) with respect to control groups (Figure 6a). In the subgroup considering BM‐MSCs, a total of 218 patients were analysed, of which 112 were treated with BM‐MSCs and 106 were without the orthobiologic treatment. The Kaplan–Meier analysis did not show a statistically significant difference between BM‐MSCs with respect to control groups (Figure 6b). In the subgroup considering PRP, a total of 194 patients were analysed, of which 97 were treated with PRP and 97 without the orthobiologic treatment. The Kaplan–Meier analysis did not show a statistically significant difference between PRP with respect to control groups (Figure 6c). Effect estimates and heterogeneity analysis for Level II analyses are reported in Supporting Information S2: Table S2.

## DISCUSSION

The main finding of this study is that orthobiologic augmentation has the potential to provide a higher survivorship over time compared to control groups for the treatment of patients affected by hip AVN. However, while BM‐MSCs, BMAC, intra‐arterial and PRP approaches held promise compared to the other treatments, the direct comparison of these approaches with respect to their controls confirmed relevant benefits only for BMAC augmentation in terms of survival from the need for THA.

The systematic review of the literature identified several orthobiologic procedures used for the treatment of AVN of the hip. The interest in regenerative therapies for AVN is rising [40], with growing evidence provided by the increasing number of RCTs, systematic reviews and meta‐analyses. Literature evidence has been analysed in the past years, with several authors supporting the overall benefit provided by orthobiologic approaches both in terms of clinical and radiological outcomes. Among the available meta‐analyses, Zhang et al. reported that the addition of bone marrow‐derived products to CD offered better clinical outcomes and lower rates of disease progression compared to CD alone in early‐stage AVN [128]. On the other hand, Jindal et al. analysed the overall efficacy of CD and BMAC in more advanced post‐collapse stages of AVN, without finding any additional benefits of BMAC when compared to CD alone [52]. The use of bone grafting associated with regenerative therapies showed satisfactory results, as reported by Migliorini et al. who conducted a Bayesian network meta‐analysis showing that the combination of CD with autologous bone grafting plus the implantation of BMAC can further decrease failure and progression to THA compared to CD alone [77]. Moreover, the meta‐analyses of Han et al. and Zhu et al. demonstrated that CD combined with PRP can improve the results of the treatment in early‐stage AVN patients, especially when combined with stem cells and bone grafts [34, 133]. While all the aforementioned literature reviews and meta‐analyses are in agreement with an overall positive outcome in terms of symptomatic relief and functional improvement, less explored is the analysis of treatment failures.

Failures in the treatment of hip AVN are a major event for these patients and can be defined as the need to undergo THA. The possibility of postponing THA is an important goal in patients affected by this condition, since they are often young and a replacement surgery could be unsatisfactory in these patients [81]. Moreover, in such young patients, undergoing THA may significantly increase the risk and frequency of revision THA procedures over their lifetime [99]. Therefore, reducing or at least postponing THA surgery in hip AVN patients is a target to ensure not only the improvement of patients' quality of life but also to reduce the cost impact of replacement procedures on the healthcare system and society [90] [12, 96, 116]. Previous meta‐analyses investigated only a few studies and focused on a specific orthobiologic treatment approach without being able to identify the best orthobiologic approach for postponing the need for THA in patients affected by hip AVN.

The present survival analysis included all studies focusing on orthobiologic augmentation for treating hip AVN and documented how various available orthobiologic treatments can delay hip replacement surgery. A multilevel analysis was conducted, considering the large number of studies and patients included. The conversion rate to THA was documented by considering all studies included in the systematic review and also by stratifying studies based on the different orthobiologic products. The overall analysis confirmed the data already provided by literature, showing an overall total estimated cumulative survivorship of 69% at 120 months of follow‐up in patients treated with orthobiologics versus 49% in patients treated with no‐orthobiologic treatments, confirming the potential of the biological augmentation approach. In addition, the analysis of the results of the different orthobiologic therapies offered interesting insights. Among the different subgroups, BM‐MSCs, BMAC, PRP and intra‐arterial treatments presented an overall longer survival compared to the control groups. In contrast, the subgroups BMAC + PRP and BMP did not show a relevant benefit compared to the control groups. Nevertheless, a further specific subanalysis for each orthobiologic approach, by comparing it with its internal control, confirmed the superiority over the control group only for BMAC treatment, while BM‐MSCs and PRP were not able to exceed the benefits provided by their control groups. In addition, the analysis of the only comparative study focusing on the intra‐arterial cell‐based treatment was not able to demonstrate the superiority of this approach over its control group. This underlines the importance of conducting specific subanalyses to achieve more robust indications of the potential of the orthobiologic augmentation approaches. Accordingly, after this multilevel analysis of the available literature, BMAC emerged as the most promising approach to address hip AVN.

This systematic review and survival analysis has a broad focus and a multilevel approach but still presents some limitations. The first one is the heterogeneity of the populations analysed, with diverse lesion stages, aetiologies and patient characteristics, which weakens the specificity of the study findings and does not allow for the conclusion for specific patients who could get the maximum benefit from the orthobiologic procedures. Moreover, AVN was evaluated with different systems, which introduces variability in the interpretation of the results. In addition, there is a lack of homogeneity among control groups. Among them were CD alone, CD plus not concentrated bone marrow aspirate, CD plus saline, CD plus bone substitutes, ESWT, colecoxib or observation. Moreover, the included articles reported different modalities of cell harvest, cell processing and cell transplantation/delivery even within each orthobiologic subgroup. Future studies should analyse patient and product characteristics that can influence the efficacy of orthobiologic therapies for the treatment of hip AVN, with direct study comparisons of the different approaches on more homogeneous patient populations. Finally, other limitations are represented by the methodology, with the lack of PROSPERO registration, quality assessment and risk of bias analysis of the included studies. Despite the aforementioned limitations, this systematic review confirms the potential of the orthobiologic approach to postpone the need for THA in patients affected by AVN. Furthermore, this study was able to preliminarily outline which orthobiologic therapies are most suitable to treat these patients, supporting the potential of BMAC as the most effective strategy. Further RCTs are needed in order to confirm these results and draw definitive conclusions on the best orthobiologic treatment, based on individual patient characteristics, and optimise the management of patients affected by hip AVN.

## CONCLUSIONS

This systematic review and survival analysis demonstrated that orthobiologics have the potential to improve survivorship in patients affected by hip AVN. In particular, the specific analysis of different orthobiologic products indicated significant benefits for BMAC augmentation in terms of survival without the need for THA, while no clear benefits were confirmed for other orthobiologics.

## AUTHOR CONTRIBUTIONS

*Conceptualisation*: Giuseppe Filardo. *Methodology*: Luca Andriolo. *Data curation*: Marco Zaffagnini, Angelo Boffa and Luca Andriolo. Writing—original draft preparation, Marco Zaffagnini and Angelo Boffa; writing—review and editing, Federico Raggi and Luca Andriolo; supervision, Giuseppe Filardo and Stefano Zaffagnini. All authors have read and agreed to the published version of the manuscript.

## CONFLICT OF INTEREST STATEMENT

S. Z. has received institutional support from Fidia Farmaceutici, Cartiheal, IGEA Clinical Biophysics, Biomet and Kensey Nash; grant support from I+ and royalties from Springer. The funders had no role in the design of the study, the collection, analysis or interpretation of data, the writing of the manuscript or the decision to publish the results. The other authors declare no conflict of interest.

## ETHICS STATEMENT

This systematic review did not involve primary data collection or human subjects, thus ethical approval was not required.

## Supporting information

Supporting information.

Supporting information.

## Data Availability

All data analysed during this study are derived from published studies and are included in this article. Additional information supporting this review is provided in the supplementary tables available with this manuscript. For any further clarifications, the corresponding author can be contacted.
