# Supplementary material for: Orthobiologic therapies delay the need for hip arthroplasty in patients with avascular necrosis of the femoral head: A systematic review and survival analysis
Source: Knee Surg Sports Traumatol Arthrosc. 2024 Nov 14;33(3):1112–27. doi: 10.1002/ksa.12532 (PMC11848991; doi:10.1002/ksa.12532)
Supplement: Supplementary file 1 — Supporting information. [file KSA-33-1112-s001.pdf]

Author Year	Level of Evidence	Patient/hip Treated	Age (range)	Staging	Etiology	Technique	F-up (range)	Failures	Conclusions	Complications
Lyu [58] BMC Musculoskelet Disord 2023	Level III Retrospective comparative study	24/29	45.8 (± 11.7)	Ficat I: 7 Ficat II: 22	Steroid: 14 Alcohol: 7 Others: 3	CD + β-TCP + grafts	62.1 m (± 17.2)	4	A single dose of PRP combined with CD and β-TCP grafts provided significant pain relief, better functional outcomes, and delayed progression in the short term compared to CD combined with β-TCP grafts.	No complications were observed.
		21/25	39.6 (± 10.8)	Ficat I: 6 Ficat II: 19	Steroid: 14 Alcohol: 5 Others: 2	CD + β-TCP grafts + PRP	59.3 m (± 14.8)	4		
Liang [52] International Orthopaedics 2023	Level IV Retrospective case series	24/24	36.4 (± 5.3)	ARCO IIA: 3 ARCO IIB: 9 ARCO IIC: 9 ARCO IIIA: 3	Idiopathic: 12 Alcohol: 6 Hormonal: 6	CD + PRP + BMMCs	41.7 m (± 3.9)	3	CD combined with autologous PRP and BMMCs grafting is a safe and effective method for the treatment of ARCO II–IIIA stage non-traumatic AVN effectively delaying or even avoiding THA.	No complications were observed.
		20/20	37.5 (± 5.3)	ARCO IIA: 3 ARCO IIB: 7 ARCO IIC: 8 ARCO IIIA: 2	Idiopathic: 9 Alcohol: 7 Hormonal: 4	CD	40.5 m (± 3.3)	8		
Jayankura [43] Clinical Orthopaedics and Related Research 2023	Level I Randomized controlled clinical trial	25/25	46 (± 10)	ARCO I: 5 ARCO II: 19 ARCO III: 1	Steroide: 6 Alcohol: 13 Tobaco: 16	CD + Osteoblastic cell implantation	17 m (± 8)	6	This study did not show any advantage in the use of autologous osteoblastic cells to improve the results of CD in patients with early-stage AVN.	One patient reported a systemic inflammatory response syndrome
		29/29	45 (± 10)	ARCO I: 3 ARCO II: 24 ARCO IV: 2	Steroid: 7 Alcohol: 24 Tobaco: 26	CD + saline	18 m (± 7)	4		No complications were observed.
Blanco [6] J Clin Med 2023	Level IV Prospective case series	8/8	48.3 (± 7.3)	ARCO 8 < IIC	Steroid: 2 Alcohol: 7	CD + BMMCs	8 y	7	There are improvements in clinical and radiological parameters, as well as a potential delay in the need for THA, but they can not be attributable alone to cellular treatment.	No complications were observed.
Hong [39] International Orthopaedics 2023	Level IV Retrospective case series	19/28	33 (21-48)	ARCO I: 3 ARCO II: 21 ARCO IIIA: 4	Alcohol: 4 Idiopathic: 5 Steroid: 10	CD + ADMSC + biochemistry artificial bone graft implantation	58.3 m (11-95)	NR	ADMSC following CD and biochemistry artificial bone graft implantation in early stage of AVN is safe and effective and could successfully repair the necrosis lesion and delay disease progression.	No complications were observed.

Baghdadi [5] J Pediatr Orthop 2023	Level IV Retrospective case series	17/23	15.8 (13.1-17.8)	Ficat I: 3<br>Ficat II: 10<br>Ficat III: 9<br>Ficat IV: 1	SCD	CD + BMAC	4.25 y (± 1.73)	3	CD-BMAC injection in pediatric patients with SCD offers significant improvement in pain and functional outcomes in the short term. Skeletal immaturity at the time of surgery was significant predictors of treatment failure.	No complications were observed.
Ulusoy [90] J Orthop Surg Res 2023	Level III Retrospective comparative study	19/19	38.4 (± 6.7)	Steinberg I: 11<br>Steinberg II: 14	Alcohol: 3<br>Idiopathic: 5<br>Trauma: 1<br>Steroid: 10	CD + BMMCs	32.2 m (± 4.1)	0	CD and the application of combined biological solutions will help protect bone tissue along with physiological remodelling and stop the progression of the disease.	No complications were observed.
		25/25	39.3 ± 6.5	Steinberg I: 10<br>Steinberg II: 9	Alcohol: 2<br>Idiopathic: 16<br>Trauma: 2<br>Steroid: 4<br>SCD: 1	CD	31.8 m (± 4)	2		
Davulcu [16] Acta Orthop Belg 2023	Level IV Retrospective case series	18/29	39.8 (± 11.7)	Ficat 2B: 25<br>Ficat 2A: 4	Steroid	CD + BMAC	13.5 m (± 3.4)	0	Autologous bone plug-sliding with CD and a BMAC application technique is effective in early-stage corticosteroid-induced AVN patients.	No complications were observed.
Zhao [104] Acta Medica Mediterranea 2022	Level I Randomized controlled clinical trial	20/20	39.5 (± 4.0)	Steinberg I: 14<br>Steinberg II: 6	NR	CD + BMMSCs	12 m	0	BMMCs combined with CD was superior to traditional CD, and was expected to provide new ideas for the treatment of AVN.	No complications were observed.
		20/20	38.4 (± 3.9)	Steinberg I: 13<br>Steinberg II: 7	NR	CD	12 m	0		
Luan [57] J Pain Res 2022	Level I Randomized controlled clinical trial	30/30	63.4 (± 11.8)	ARCO I: 3<br>ARCO II: 11<br>ARCO III: 16	Non-traumatic	PRP	12 m	2	This study supported the effectiveness and safety of both the PRP injection and ESWT in treating AVN patients. For symptomatic patients with ONFH, intra-articular PRP injection appeared superior to ESWT in pain relief and functional improvement.	No complications were observed.
		30/30	61.6 (± 11.8)	ARCO I: 3<br>ARCO II: 13<br>ARCO III: 14	Non-traumatic	ESWT	12 m	2		
Gómez-Barrena [25] Bone Joint Res. 2022	Level IV Retrospective case series	22/22	43 (± 10.6)	ARCO II	Idiopathic: 20<br>Steroid: 2	CD + BM-hMSCs	12 m	3	Bone regeneration was observed in AVN 3 m after expanded autologous BM-hMSC injection, and the volume and location of regeneration indicated the success of the therapy.	No complications were observed.

Zhang [103]<br>Front Cell Dev Biol.<br>2022	Level I<br>Randomized controlled clinical trial	26/26	46.2<br>(± 2.8)	ARCO I: 8<br>ARCO II: 18	NR	LPCs perfusion	NR	NR	LPCs possess a superior vascularization capacity in both autonomous and paracrine manner, indicating that autologous LPCs perfusion via the medial circumflex artery is an effective therapy for AVN.	NR
		8/8	48.1<br>(± 6.7)	ARCO I: 2<br>ARCO II: 6	NR	Saline perfusion	NR	NR		
Yoshizawa [102]<br>Indian J Orthop<br>2022	Level IV<br>Retrospective case series	46/60	41.1<br>(± 12.9)	JIC<br>Stage 1: 7<br>Stage 2: 19<br>Stage 3A: 20<br>Stage 3B: 9<br>Stage 4: 5<br>Type C1: 13<br>Type C2: 47	Steroid 48<br>Alcohol 7<br>Idiopathic 5	CD + CABMAT	NR	NR	CABMAT was found to be a useful hippreserving surgery that had little effect on conversion THA.	NR
Yang [98]<br>Eur Rev Med Pharmacol Sci<br>2022	Level IV<br>Retrospective case series	18/26	42.6<br>(± 5.8)	ARCO IIA: 4<br>ARCO IIB: 9<br>ARCO IIC: 13	Alcohol: 5<br>Idiopathic: 5<br>Trauma: 1<br>Steroid: 7	CD + hUC-MSCs	18.6 m<br>(± 4.5)	NR	Robot-assisted CD combined with hUC-MSC transplantation is a feasible and relatively safe method for the treatment of AVN.	No complications were observed.
Tomaru [87]<br>Cureus<br>2022	Level III<br>Retrospective comparative study	232/387	40.1<br>(14.3-77.2)	JIC<br>Stage 1: 106<br>Stage 2: 122<br>Stage 3A: 101<br>Stage 3B: 45<br>Stage 4: 13<br>Type A: 4<br>Type B: 32<br>Type C1: 168<br>Type C2: 183	Steroid: 280<br>Alcohol: 76<br>Idiopathic: 31	CD + CABMAT	8.2 y<br>(2.1-17.6)	94	Collapse rates were significantly higher for stage 1 AVN; for collapse stages, the THA conversion rates were significantly lower in the CABMAT group than in the observation group. Therefore, observation and CABMAT are recommended for AVN of stage 1 and for AVN of higher stages, respectively.	1 subtrochanteric femur fracture , which required open reduction and internal fixation.
		106/171	48.9<br>(14.3-84.4)	JIC<br>Stage 1: 47<br>Stage 2: 51<br>Stage 3A: 42<br>Stage 3B: 22<br>Stage 3C: 9<br>Type A: 1<br>Type B: 24<br>Type C1: 57<br>Type C2: 89	Steroid: 137<br>Alcohol: 22<br>Idiopathic: 12	Observation	6 y<br>(2.0-19.2)	71		No complications were observed.

Sugaya [83]<br>EJOST<br>2022	Level IV<br>Prospective case series	16/26	38.0<br>(25-50)	JIC<br>Stage 1: 1<br>Stage 2: 6<br>Stage 3A: 18<br>Type B: 3<br>Type C1: 16<br>Type C2: 7	Steroid: 14<br>Alcohol: 1<br>Trauma: 1	CD + CABMAT + LIPUS	48 m<br>(30-56)	NR	Treatment with CABMAT combined with 3-month LIPUS stimulation was safe, and only one hip underwent THA	No complications were observed.
Sadat-Ali [77]<br>JEO<br>2022	Level IV<br>Prospective case series	63/63	25.9<br>(± 5.5)	Ficat I: 5<br>Ficat II: 47<br>Ficat III: 11	SCD	CD + Osteoblastic cell implantation	40 m<br>(± 8.9)	NR	This study give credence to our earlier short follow-up results showing that osteoblast transplantation is a good approach in healing of the avascular lesions in SCD.	No complications were observed.
Hoogervorst [40]<br>J Bone Joint Surg Am.<br>2022	Level III<br>Retrospective comparative study	40/61	33.4<br>(± 10.3)	ARCO I: 9.8%<br>ARCO II: 73.8%<br>ARCO III: 16.4%	Steroid: 82.0%<br>Alcohol: 14.8%<br>Other: 3.3%	CD + BMAC	28.9 m<br>(± 28.1)	16	CD with BMAC grafting is a safe treatment option in the care of patients with AVN. Independent statistically significant predictors of progression-free survival or conversion to THA are BMI ≥ 30 and the extent of AVN.	One femoral neck stress fracture requiring open reduction and internal fixation.
		NR/24	39.8<br>(± 12.6)	ARCO I: 25.0%<br>ARCO II: 66.7%<br>ARCO III: 4.2%<br>ARCO IV: 4.2%	Steroid: 82.6%<br>Alcohol: 8.7%<br>Other: 8.7%	CD	44.5 m<br>(± 46.7)	13		No complications were observed.
		NR/19	39.4<br>(± 11.3)	ARCO I: 21.1%<br>ARCO II: 79.0%	Steroid: 79.0%<br>Alcohol: 15.8%<br>Other: 5.3%	CD + PMMA Augmentation	54.2 m<br>(± 43.4)	7		No complications were observed.
Tomaru [88]<br>J Rural Med.<br>2021	Level III<br>Retrospective comparative study	NR/33	35.1<br>(22.6–53.5)	JIC<br>Stage 1: 15<br>Stage 2: 13<br>Stage 3A: 2<br>Stage 3B: 3<br>Type A: 1<br>Type B: 2<br>Type C1: 16<br>Type C2: 14	Steroid-induced AVN in SLE	CD + CABMAT	5.9 y<br>(2.0–14.5)	11	Based on the collapse and THA conversion rates, conservative therapy was more favorable for stage 1, whereas CABMAT was more favorable for stage 3.	No complications were observed.
		NR/33	35.7<br>(20.5–57.1)	JIC<br>Stage 1: 15<br>Stage 2: 13<br>Stage 3A: 2<br>Stage 3B: 3<br>Type A: 1<br>Type B: 2<br>Type C1: 16<br>Type C2: 14		Conservative	8.7 y<br>(2.1–24.8)	15		No complications were observed.

Palekar [69]<br>Orthopedics<br>2021	Level IV<br>Case series	15/24	32<br>(21-61)	ARCO II: 15<br>ARCO III: 9	Idiopathic: 8<br>Steroid: 5<br>Alcohol+Steroid: 1<br>Trauma: 1	CD + ABMDO	4.2 y<br>(1.5-7)	0	The short-term and log-term results of ABMDO treatment along with routine procedures have been satisfactory in patients with early AVN	No complications were observed.
Ma [59]<br>Orthop Surg.<br>2021	Level IV<br>Retrospective case series	17/30	36.8<br>(± 7.9)	JIC<br>Stage 2: 6<br>Stage 3A: 14<br>Stage 3B: 10<br>Type B: 3<br>Type C1: 3<br>Type C2: 24	Steroid: 7<br>Tobacco: 16<br>Alcohol: 12	CD + BMMCs + Zolendronate	69.1 m<br>(± 20.5)	6	CD with local administration of zoledronate and enriched BMMCs could relieve the pain, delay the progression of collapse, and postpone the time of THA. ARCO staging and JIC type and corticosteroid exposure were found to be relevant factors affecting hip preservation.	Four patients complained about general weakness and muscle aches within 2–3 days post-operation
Khan [47]<br>Ann R Coll Surg Engl.<br>2021	Level IV<br>Retrospective case series	13/14	41.1<br>(21-70)	Ficat I–II	Idiopathic: 5<br>Alcohol: 4<br>Trauma: 3<br>Steroid: 2	CD + BMAC	18 m	6	For patients with Ficat stage I or II of the femoral head, early results suggest that CD combined with BMAC appears to be a viable treatment option to provide symptomatic relief, particularly in patients with no comorbidities or smaller zones of head involvement	No complications were observed.
Hernigou [34]<br>Morphologie<br>2021	Level III<br>Retrospective comparative study	23/23	45<br>(22-63)	Steinberg I-II: 15	Trauma	BMAC	15 y<br>(10-20)	NR	The present study has demonstrated encouraging effects of cell therapy in early post-traumatic AVN and provides another choice for treatment in stages I to II.	No complications were observed.
		23/23	47<br>(24-65)	Steinberg III-IV: 8	Trauma	THA	15 y<br>(10-20)	NR		No complications were observed.
Goto [27]<br>J Orthop<br>2021	Level IV<br>Prospective case series	10/10	31.7<br>(20–48)	JIC<br>Stage 3A: 6<br>Stage 3B: 4<br>Type C1: 1<br>Type C2: 9	Steroid: 4	CD+ expanded MSCs + vascularised bone grafts + β-TCP (OSferion®)	10 y	2	BMMCs in combination with vascularised bone grafts could be an effective treatment for post-collapse AVN.	No complications were observed.

Gómez-Barrena [26]<br>J. Clin. Med.<br>2021	Level IV<br>Prospective case series	22/22	43.1<br>(± 10.9)	ARCO IIA: 15<br>ARCO IIB: 6<br>ARCO IIC: 1	Idiopathic: 11<br>Steroid: 5<br>Alcohol: 1<br>SCD: 2<br>Alcohol+Steroid: 1<br>Thalassemia: 1<br>Octreotide: 1	CD + expanded MSCs	5 y<br>(12m-6y)	4	Expanded MSCs implantation was safe. Early efficacy was confirmed in 80% of cases under protocol at 2 years. At 5 years, the overall results were maintained and 19% converted to THA, all in the first year.	1 deep venous thrombosis (1m)<br>1 open reduction and internal fixation of controlaterl femure (3m)<br>1 acute arthritis that needed a THA (8m)<br>1 upper gastrointestinal bleeding (12m)
Boontanapibul [7]<br>J Arthroplasty<br>2021	Level III<br>Retrospective comparative study	40/50	38<br>(± 13)	ARCO I: 6<br>ARCO II: 39<br>ARCO IIIA: 5	Steroid: 21<br>Chemotherapy+Steroid: 9<br>Idiopathic: 9<br>Alcohol: 8<br>SCD: 3	CD + BMAC	35 m<br>(± 20)	NR	Addition of BMAC had more reliable outcomes than isolated CD for precollapse AVN if the combined necrotic angles were <250°.	NR
		26/33	43<br>(± 10)	ARCO I: 6<br>ARCO II: 25<br>ARCO IIIA: 2	Idiopathic: 8<br>Steroid: 13<br>Alcohol: 4<br>SCD: 3<br>Chemotherapy+Steroid: 5	CD	38 m<br>(± 26)	NR		
Li [51]<br>Stem Cell Res Ther<br>2021	Level I<br>Randomized controlled clinical trial	17/22	35.4<br>(± 11.1)	Ficat I: 1<br>Ficat II: 19<br>Ficat III: 2	Steroid: 8<br>Alcohol: 5<br>Idiopathic: 9	CD + BBC + ABR grafting	2 y<br>(NR-5)	1	The survivorship of the femoral head is higher for the patients receiving BBC and ABR with ACD.	No complications were observed.
		23/29	39.4<br>(± 10.4)	Ficat I: 1<br>Ficat II: 20<br>Ficat III: 6<br>Ficat IV: 2	Steroid: 3<br>Alcohol: 9<br>Idiopathic: 17	CD + β-TCP + ABR grafting		5		
Yoon [100]<br>Clin Orthop Surg<br>2021	Level IV<br>Prospective case series	15/18	43.8<br>(20-59)	ARCO II: 14<br>ARCO III: 4	Steroid: 2<br>Alcohol: 7<br>Caisson Disease: 1<br>Idiopathic: 6	CD + ADMSC	24 m	3	Culture-expanded ADMSC implantation is a viable option for AVN treatment without adverse events.	No complications were observed.
Kuroda [49]<br>Regen Med<br>2021	Level III<br>Retrospective comparative study	49/78	42.5<br>(± 14.8)	JIC<br>Stage 1: 17<br>Stage 2: 32<br>Type C1: 18<br>Type C2: 31	Steroid: 36<br>Steroid+Alcohol: 1<br>Alcohol: 11<br>Idiopathic: 1	CD + rhFGF-2	24 m	NR	rhFGF-2 treatment safely increases the joint preservation time with clinical efficacy and radiological bone regeneration. Thus may be one of the viable therapeutic options for early-stage AVN.	Unrelated complications
		49/78	43.8<br>(± 15.3)	JIC<br>Stage 1: 15<br>Stage 2: 34<br>Type C1: 14<br>Type C2: 35	Steroid: 36<br>Steroid+Alcohol: 1<br>Alcohol: 11<br>Idiopathic: 1	No treatment	24 m	NR		Unrelated complications

Houdek [41] Bone Jt Open 2021	Level IV Case series	22/35	43 (± 12)	Steinberg I: 4 Steinberg II: 31	Steroid	CD + BMAC + PRP	7 y (5-8)	10	At seven years, CD augmented with BMAC and PRP provided a 67% survivorship free from THA in patients with corticosteroid-induced AVN. Ideal candidates for this procedure are patients with low preoperative Kerboul angles and can stop corticosteroid treatment prior to CD.	1 dead for comorbidities
Rocchi [75] Hip International 2020	Level IV Retrospective case series	52/52	NR	ARCO I: 1 ARCO II: 21 ARCO III: 26 ARCO IV: 4	NR	CD + PRP + BMAC	50.6 m (± 8.7)	20	CD associated with bone allograft combined with PRP and BMSCs is a procedure with low morbidity that allows us to avoid or delay the need for THA. The indication of choice is early ARCO grade necrosis before collapse of the articular surface and the size has to be less than 1/3 of the femoral head.	NR
Li [50] Stem Cell Res Ther 2020	Level I Randomized controlled clinical trial	17/21	34.1 (± 8.0)	Ficat II: 11 Ficat III: 10	Steroid: 10 Alcohol: 6 Idiopathic: 5	CD + BG + BBC	10 y	NR	Autologous BBC in combination with CD was more effective than the use of CD alone. The preoperative Ficat stage was an independent risk factor for predicting the postoperative survival rate. Ficat stage III hips had a higher risk for progression.	No complications were observed.
		14/20	38.2 (± 8.1)	Ficat II: 11 Ficat III: 9	Steroid: 9 Alcohol: 5 Idiopathic: 6	CD + BG	10 y	NR		
Hauzeur [31] Clin Orthop Relat Res 2020	Level II Single blinded randomized therapeutic study	26/26	50 (± 12)	ARCO I: 10 ARCO II: 16	Steroid: 19 Alcohol: 11 Idiopathic: 1 Other: 5	CD + BMAC	36 m (35-38)	NR	Given the fact that osteoblastic cells were no more effective than BMAC for the treatment of AVN, and considering the large cost associated with osteoblastic cell expansion cultures, we can not recommend the use of osteoblastic cells for this indication.	No complications were observed.
		27/27	51 (± 10)	ARCO I: 10 ARCO II: 17	Steroid: 16 Alcohol: 7 Idiopathic: 8 Others: 3	CD + Osteoblastic cell implantation		NR		One severe pyrexia

Wu [93]<br>BMC Musculoskelet Disord.<br>2020	Level IV<br>Retrospective case series	30/30	30.6<br>(± 5.1)	ARCO II	NR	CD + hBMSCs	24 m	NR	The quality of implanted stem cells is closely related to treatment efficacy and determines whether the defective self-repair in the necrotic area can be corrected to enhance repair and thus achieve the desired therapeutic outcome.	NR
Martinot [65]<br>Int Orthop<br>2020	Level III<br>Retrospective comparative study	24/24	41.0<br>(± 9.5)	Arlet and Ficat Classification<br>Stage 1: 1<br>Stage 2: 22<br>Stage 3: 1	Alcohol: 6<br>Chronic disease: 2<br>Steroid: 5<br>Tobacco: 6<br>Transplant: 2	CD	64 m<br>(± 64.5)	13	CD is an interesting non-invasive technique to preserve the native hip after AVN. Reinjection of BM and/or BMP improved CD hip survival.	No complications were observed.
		25/25	37.4<br>(± 10.1)	Ficat I: 6<br>Ficat II: 17<br>Ficat III: 2	Chronic disease: 4<br>Steroid: 10<br>Tobacco: 5	CD + BM + rhBMP7		7		No complications were observed.
		43/43	43.2<br>(± 10.9)	Ficat I: 3<br>Ficat II: 37<br>Ficat III: 3	Alcohol: 6<br>Chronic disease: 7<br>SLE: 3<br>Steroid: 21<br>Tobacco: 14<br>Transplant: 2	CD + BM		16		One postoperative subtrochanteric fracture of the femur requiring fixation by screw plate.
Hernandez [33]<br>Clin Orthop Surg.<br>2020	Level IV<br>Prospective case series	10/18	37.8<br>(± 10.9)	ARCO I: 11<br>ARCO II: 6	Alcohol: 6<br>Steroid: 12	CD + BMAC + TCP	68.9 m<br>(± 15)	9	CD combined with implantation of BMAC and TCP will not prevent radiographic progression of early stage AVN.	One subtrochanteric fracture after surgery, requiring osteosynthesis with an intramedullary nail.
Döring [17]<br>Stem Cells Dev.<br>2020	Level III<br>Retrospective comparative study	10/14	16.9<br>(8.5-25.8)	Ficat I-II	Chemotherapy: 6<br>Steroid: 3<br>Osteochondrosis dissecans: 1	CD + Cultured MSCs	3.1 y<br>(1.6-5.8)	1	In the long-term f-up, MSC treatment showed a superior radiological outcome, compared with the conventional CD alone. Clinical improvement of pain and mobility of the affected joints was not significantly different between the two study groups.	No complications were observed.
		11/13	17.9<br>(13.5-27.5)		Chemotherapy: 11	CD	2.0 y<br>(1.5–8.5)	0		No complications were observed.

Ying [99]<br>Journal of Orthopaedic Research<br>2020	Level II<br>Prospective comparative study	17/17	38.7<br>(± 8.5)	NR	NR	Porous tantalum rod implantation + PBSCs	36 m	11	The combination treatment of porous tantalum rod implantation and intra-arterial infusion of PBSCs is not an ideal choice to treat AVN patients with megalgia symptoms and inflammation-induced osteoclastogenesis.	NR
		20/20	37.3<br>(± 8.2)	NR	NR	Celecoxib	36 m	13		
Pan [70]<br>Stem Cell Res Ther<br>2020	Level IV<br>Retrospective case series	35/47	39.0<br>(± 12.5)	ARCO IA: 5<br>ARCO IB: 8<br>ARCO IC: 6<br>ARCO IIA: 9<br>ARCO IIB: 12<br>ARCO IIC: 7	Steroid: 6<br>Alcohol: 6<br>Idiopathic: 23	Auto-PBSCs	53.9 m<br>(± 21.0)	NR	The results suggest that intra-arterial infusion of auto-PBSCs prolongs femoral head survival. Age, BMI, HHS, and necrotic volume can influence the efficacy of this intervention.	No complications were observed.
Grassi [28]<br>International Orthopaedics<br>2020	Level IV<br>Prospective case series	22/30	42<br>(23-60)	Ficat I: 6<br>Ficat IIA: 12<br>Ficat IIB: 12	Idiopathic: 12<br>Steroid: 10	CD + PRP	5 y	16	CD combined with PRP could be indicated as a treatment for the I and IIA stages of AVN, as it significantly reduces joint pain and delays THA. Should be avoided in AVN related to cortisone therapy because only a few benefits have been proven.	5 fever
Aggarwal [2]<br>The Journal of Arthroplasty<br>2020	Level I<br>Randomized controlled clinical trial	19/25	38.2<br>(± 10.4)	Ficat I: 7<br>Ficat II: 18<br>ARCO I: 7<br>ARCO II: 18	Alcohol: 16<br>Idiopathic: 14<br>Steroid: 10	CD + PRP	64.3 m<br>(54-72)	NR	PRP use after CD provides significant pain relief, better midterm functional outcome, retards the progression, and enhances the survivorship free from reoperation for THA and femoral head collapse in early stages of AVN than CD alone.	No complications were observed.
		21/28	35.2<br>(± 12.5)	Ficat I: 6<br>Ficat II: 22<br>ARCO I: 6<br>ARCO II: 22		CD	63.7 m<br>(56-72)	NR		No complications were observed.
Majeed [29]<br>Acta Medica Iranica<br>2020	Level II<br>Prospective comparative study	24/16	34.6<br>(22-46)	Ficat I: 3<br>Ficat IIA: 7<br>Ficat IIB: 6	Non-traumatic	CD + PRP	12 m	0	The addition of PRP to CD for pre-distorted stages of the head of the femur had resulted in improved pain alleviation and functional results and had slowed the disease progression in the one year of f-up.	NR
		24/16		Ficat I: 4<br>Ficat IIA: 6<br>Ficat IIB: 6		CD	12 m	0		NR

Xian [94]<br>The Journal of Arthroplasty<br>2019	Level I<br>Randomized controlled clinical trial	26/26	28.3<br>(22-46)	ARCO IIA: 6<br>ARCO IIB: 3<br>ARCO IIC: 2<br>ARCO IIIA: 8<br>ARCO IIIB: 4<br>ARCO IIIC: 1	Trauma	CD + PRP + Incorporated autologous granular bone graft	44.9<br>(± 1.7)	3	PRP-incorporated autologous granular bone grafting appears to be an effective and safe method for treatment in ARCO stages II to III of post-traumatic AVN, which could achieve better clinical and radiological results compared with autologous granular bone grafting alone.	No complications were observed.
		22/22	29.6<br>(22-46)	ARCO IIA: 6<br>ARCO IIB: 2<br>ARCO IIC: 1<br>ARCO IIIA: 7<br>ARCO IIIB: 5<br>ARCO IIIC: 1	Trauma	CD + Incorporated autologous granular bone graft	46.2<br>(± 2.2)	5		No complications were observed.
Tomaru [89]<br>BMC Musculoskelet Disord.<br>2019	Level IV<br>Retrospective case series	44/80	42.2<br>(16.3-70.5)	ARCO I: 12<br>ARCO II: 31<br>ARCO III: 32 ARCO IV: 5	Alcohol: 19<br>Idiopathic: 5<br>Steroid: 56	CD + CABMAT	12 y<br>(10-15.4)	25	On the basis of our long-term findings, the minimally invasive and feasible CABMAT therapy can be utilized as one of a joint-preserving treatment for AVN.	No complications were observed.
Mardones [63]<br>Stem Cells Cloning.<br>2019	Level IV<br>Case series	5/5	41.2<br>(23-59)	Ficat II: 4<br>Ficat III: 1	Hypothyroidism: 1<br>Idiopathic: 2<br>Insulin resistance: 1<br>SLE: 1	CD + Expanded MSCs	19-54 m	0	The hip function after MSC-based therapy was significantly improved and pain intensity markedly reduced. As a corollary, no patient required THA.	No complications were observed.
Emadedin [19]<br>Cytotherapy<br>2019	Level IV<br>Case series	9/9	26<br>(23-34)	Steinberg III: 9	Steroid: 7<br>Idiopathic: 2	CD + Autologous bone marrow derived CD133+ cells	12 m	0	A single bone marrow derived CD133+ cell injection into the necrotic site of the femoral head during CD is safe and effective in providing significant, clinically relevant pain relief and patients could do more activity over 2, 6 and 12 months.	No complications were observed.
Talathi [86]<br>J Clin Orthop Trauma<br>2018	Level IV<br>Retrospective case series	28/43	40.5<br>(22-61)	ARCO IA: 3<br>ARCO IB: 4 ARCO IC: 1 ARCO IIA: 8 ARCO IIB: 3 ARCO IIC: 9	Alcohol: 5<br>Idiopathic: 2 Multiple causes: 3<br>SCD: 3<br>Steroid: 15	CD + BMAC	16 m<br>(NR-24)	3	For patients with ARCO stage I or II of the femoral head, hip CD combined with BMAC appears to be a viable treatment option to provide symptomatic relief and perhaps arrest progression of this disease.	One post-operative deep venous thrombosis

Liu [56]<br>International Orthopaedics<br>2018	Level IV<br>Retrospective case series	148/192	38.3<br>(13-78)	ARCO I: 6<br>ARCO IIA: 25<br>ARCO IIB: 74<br>ARCO IIC: 67<br>ARCO III: 21	Alcohol: 81<br>Idiopathic: 52<br>Steroid: 59	CD + BMMCs	34.9 m<br>(3-60)	NR	Disease type is an important risk factor for autologous BMMCs combined with CD, and the degree of lateral pillar necrosis is a significant reference index for prognosis evaluation in early stage of AVN.	One bleeding and haematoma at puncture point
Kang [45]<br>Stem Cell Res Ther<br>2018	Level III<br>Retrospective comparative study	50/53	46.0<br>(± 9.3)	ARCO I: 1<br>ARCO II: 29<br>ARCO III: 19 ARCO IV: 4	Alcohol: 19<br>Idiopathic: 24<br>Other: 5<br>Steroid: 5	BMAC + CD	4.5 y<br>(3-10)	15	implantation of autologous MSCs into the femoral head in the early stage of AVN lowers the THA conversion rate. However, the progression of the ARCO stage is not affected by this treatment.	No complications were observed.
		50/53	47.3<br>(± 9.7)	ARCO I: 1<br>ARCO II: 29<br>ARCO III: 19 ARCO IV: 4	Alcohol: 19<br>Idiopathic: 24<br>Other: 5<br>Steroid: 5	CD	4 y<br>(3-10) y	26		
Hernigou [36]<br>International Orthopaedics<br>2018	Level II<br>Prospective comparative study	125/125	36<br>(18-54)	Steinberg I: 138<br>Steinberg II: 112	Steroid	CD + BMAC	25<br>(20-30)	NR	BMCs implantation in the necrotic lesion could be an efficacious treatment of early stages AVN to delay disease progression, reduce the incidence of collapse, and avoid THA even in long-term f-up.	NR
		125/125				CD		NR		
Hernigou [38]<br>International Orthopaedics<br>2018	Level I<br>Randomized controlled clinical trial	30/30	NR	Steinberg I: 28<br>Steinberg II: 32	NR	CD + BMAC under fluoroscopy	6 m<br>(minimum)	NR	Computer navigation may be safely used in a basic procedure for injection of stem cells. It reduced radiation exposure and better repair with accurate placement of trocars.	No complications were observed.
		30/30			NR	CD + BMAC under computer-assisted surgery	6 m<br>(minimum)	NR		
Hauzeur [30]<br>International Orthopaedics<br>2018	Level I<br>Randomized controlled clinical trial	19/23	48.0<br>(± 2.8)	ARCO III	Steroid: 12<br>Alcohol: 8<br>Idiopathic: 1<br>SCD: 2	CD + BMAC	24 m	15	Implantation of BMAC after CD did not produce any improvement of the evolution of AVN stage 3.	1 pain at the great trochanter<br>2 pain at iliac crest
		19/23	49.7<br>(± 3.2)	ARCO III	Steroid: 13<br>Alcohol: 7<br>Idiopathic: 3	CD + saline	24 m	23		3 pain at the great trochanter
D'Ambrosi [15]<br>Joints<br>2018	Level IV<br>Prospective case series	16/26	41.9<br>(± 9.8)	Ficat I: 2<br>Ficat II: 8<br>Ficat III: 10<br>Ficat IV: 6	Idiopathic: 8<br>Alcohol: 3<br>HIV: 2<br>Steroid: 3	CD + PRP + BMAC + Syntethic bone graft	4.2 y<br>(± 1.8)	12	The technique is safe and good preliminary results were obtained in patients with early stages of AVN, the risk of failure was higher in patients at stage III and IV, as an irreversible damage of the joint is already in place.	No complications were observed.

De Rojas [76]<br>Clin Transl Oncol<br>2018	Level IV<br>Prospective<br>case series	2/4	19.5<br>(19-20)	Ficat II: 1<br>Ficat III: 1<br>ARCO IV:2	Steroid	CD + MSCs	4 y	0	Our preliminary results suggest that autologous MSCs can be considered as a novel treatment for children and young adults with AVN by leukaemia. It may avoid THA and improve quality of life of leukaemia survivors.	No complications were observed.
Sadat-Ali [78]<br>Int J Stem Cells<br>2017	Level IV<br>Prospective<br>case series	11/11	20.2<br>(± 3.9)	Ficat I-II	SCD	CD + Osteoblast implantation	6 m	0	The positive scenario of the procedure is that it will give the patients, pain free movements and better quality of life and delay the collapse of the head of femur requiring early THA.	No complications were observed.
Einhorn [18]<br>Seminars in Arthroplasty<br>2017	Level IV<br>Prospective<br>case series	52/66	40<br>(17-67)	ARCO I-II	Alcohol: 9<br>Alcohol+Steroid: 18<br>Chemotherapy: 1<br>Idiopathic: 7<br>SCD: 6<br>Steroid: 20<br>Trauma: 5	CD + BMAC	2 y	NR	CD-BMAC may significantly improve patient function, reduce stiffness, and alleviate pain. CD-BMAC may effectively delay disease progression in patients with early stage AVN.	No complications were observed.
Shi [82]<br>Medicine (Baltimore)<br>2017	Level III<br>Retrospective<br>comparative<br>study	46/66	NR	ARCO IIA: 12<br>ARCO IIB: 36<br>ARCO III: 18	Steroid<br>Alcohol<br>Idiopathic	CD + BG + rhBMP-2	18<br>(12-25)	NR	rhBMP-2 has osteoinductive property and might serve as an adjuvant therapy in the surgical treatment of AVN. However, the incidence of HO formation might increase when used in high doses.	1 lateral femoral cutaneous nerve lesion
		48/75	NR	ARCO IIA: 16<br>ARCO IIB: 38<br>ARCO IIIA: 21	Steroid<br>Alcohol<br>Idiopathic	CD + BG	16<br>(13-25)	NR		2 lateral femoral cutaneous nerve lesion
Hyodo [42]<br>The Journal of Hip Surgery<br>2017	Level IV<br>Retrospective<br>case series	123/213	40<br>(14-70)	JIC<br>Stage 1: 47<br>Stage 2: 66<br>Stage 3A: 58<br>Stage 3B: 34<br>Stage 4: 8<br>Type A: 10<br>Type B: 9<br>Type C1: 78<br>Type C2: 116	Steroid: 144 Alcohol: 46<br>Idiopathic: 23	CABMAT	60.5 m<br>(24-116)	NR	The most effective predictive factor for THA conversion was the disease type C2. It can predict conversion to THA and suggest which patients with idiopathic AVN are appropriate for CABMAT treatment.	No complications were observed.

Pilge [73] OrthoRev 2016	Level II Prospective comparative study	10/10	38.4 (15-58)	ARCO 2: 12 ARCO 3: 6 ARCO 4: 2	Steroid: 5 Chemotherapy: 6 Idiopathic: 8 Smoke: 1	CD + iloprost iv.	30.6 m (4-69)	NR	An improvement in clinical scores was shown in treatment group, but not in control group. 2 patients in treatment group and 4 in control group were treated with THR.	3 patients had flush symptoms and 2 patients complained of a mild headache during infusion.
		10/10				CD combined with BMC + iloprost iv.		NR		
Chen [10] Molecular Medicine Reports 2016	Level IV Case series	9/9	41.1 (28-51)	ARCO II: 5 ARCO IIIa: 4	Steroid: 6 Alcohol: 2 Idiopathic: 1	Intra−arterial injection of hUC-MSCs	24 m	NR	Intra-arterial infusion of hUC−MSCs promote the repair and regeneration in the condition of bone necrosis.	No specific complication.
Gao [22] Nature Scientific Report 2016	Level IV Case series	51/51	16.3 (11.4-18.1)	ARCO I: 3 ARCO II: 21 ARCO III: 27	Femoral neck fractures	CD + implantation of BMC and rhBMP-2	6.8 y	0	The combination of CD + implantation of BMC and rhBMP-2 provided beneficial effects for hips affected by early- to middle-stage osteonecrosis after femoral neck fractures in children and adolescent. 1 patient required THA.	No complications were observed.
Gianakos [24] HSS Journal 2016	Level III Retrospective comparative study	40/40	43	Ficat I: 3 Ficat IIa: 25 Ficat IIb: 1	Steroid: 20 Idiopathic: 15 Anticoagulation: 4 Trauma: 1	Bisphosphonate therapy alone	25.3 m	NR	This study demonstrates no significant difference in clinical outcomes between the two treatment groups. Treatment with bisphosphonate + CD + BMC show a similar chance of progressing in AVN compared to hips treated with bisphosphonate alone.	NR
		22/22	38	Ficat I: 1 Ficat IIa: 13 Ficat IIb: 6	Steroid: 8 Idiopathic: 12 Anticoagulation: 2	Bisphosphonate therapy in combination with CD and BMC	22.7 m	NR		
Mishima [66] Eur J Orthop Surg Traumatol 2016	Level IV Case series	14/22	40 (20–58)	JOA B: 2 JOA C1: 10 JOA C2: 10	Steroids: 15 Trauma: 3 Alcohol: 2 Idiopathic: 2	CD with BMC and LIPUS	26 m (24–30)	NR	BMC plus LIPUS offer a safe and effective treatment of AVN.	No complications were observed.
Yan [97] Current Orthopedic Practice 2016	Level III Retrospective comparative study	42/42	37.2	ARCO I: 2 ARCO II: 40	Steroid: 29 Alcohol: 13	CD alone	26.3 m	NR	BMC transplantation in addition to CD relives articular pain and delay the progression of early AVN.	No complications were observed.
		44/44	39.6	ARCO I: 3 ARCO II: 41	Steroid: 28 Alcohol: 16	CD + BMC implantation		NR		
Pepke [71] Orthopedic Reviews 2016	Level I Randomized controlled clinical trial	NR/14	44.5	ARCO II: 25	Chemotherapy: 2 Immunosuppressive therapy: 4	CD alone	24 m	NR	No significant benefit from the additional injection of BMC in the short term.	No complications were observed.
		NR/11	44.3			CD + BMC implantation		NR		

Samy [79]<br>Indian J Orthop<br>2016	Level IV<br>Case series	30/40	36.7<br>(20-48)	Ficat IIb: 16<br>Ficat III: 24	Steroid: 15<br>Idiopatic: 20<br>Trauma: 5	Drilling of necrotic area and filled with a composite of bone graft mixed with PRP	41.4 m<br>(36-50)	NR	The use of bone graft mixed with PRP improve the reparable capacity of necrotic area.	No complications were observed.
Cruz-Pardos [13]<br>Hip Int<br>2016	Level III<br>Retrospective comparative study	NR/19	36.7<br>(20-68)	Ficat I: 5<br>Ficat II: 11	Steroid: 6<br>Alcohol: 5<br>Idiopathic: 6<br>Anticoagulation: 1<br>Other: 1	CD alone	45 m<br>(24-171)	NR	No significant radiologic and clinical differences between outcome of CD plus BMC and CD alone.	No complications were observed.
		NR/41	42.6<br>(23-70)	Ficat I: 8<br>Ficat II: 33	Steroid: 14<br>Alcohol: 3<br>Idiopathic: 12<br>HIV: 8	CD combined with BMC grafting into the core tract		NR		
Kuroda [48]<br>Int Orthop<br>2015	Level IV<br>Case series	10/10	39.8<br>(29-53)	JOA<br>Stage 1: 1<br>Stage 2: 9<br>Type A: 2<br>Type C1: 1<br>Type C2: 7	Steroid: 18<br>Alcohol: 6	CD with single local administration of 800 µg of rhFGF-2-impregnated gelatin hydrogel	12 m	NR	Stage progression and collapse did not occur in nine cases, with significant improvement of clinical scores by one year postoperatively. Computed tomography confirmed bone regeneration in the femoral heads.	1 adverse event related to surgery (headache due to spinal anesthesia).
Persiani [72]<br>Acta Orthop Belg<br>2015	Level IV<br>Case series	29/31	34<br>(26-53)	Steinberg I: 11<br>Steinberg II: 16<br>Steinberg III: 2<br>Steinberg IV: 2	Steroid: 18<br>Alcohol: 6<br>Idiopathic: 7	CD + BMC implantation	37 m<br>(23-48 m)	NR	25 hips showed relief of symptoms and resolution of the osteonecrosis at magnetic resonance imaging. This technique is effective in delaying THA in early stages.	No complications were observed.
Gao [23]<br>J Nanomater<br>2015	Level IV<br>Case series	12/12	NR	Ficat II: 12	NR	Implantation of novel nanoscaledCD rod + umbilical cord MSC	12 m	NR	This combined treatment provides significant improvements of HHS without failures in the first year of f-up, suggesting that is effective for the treatment of early AVN	No complication was observed.

Aoyama [3]<br>Arch Phys Med Rehabil<br>2015	Level IV<br>Case series	10/10	31.7<br>(20-48)	JOA IIIA: 6<br>JOA IIIB: 4	Steroid: 4<br>Idiopathic: 6	Cultured bone-marrow derived MSC transplantation augmented by vascularized bone grafting	12 m	0	Cultured bone-marrow derived MSC transplantation in individuals with AVN provides significant improvements on external rotation, extensor and abductor muscle strength, and physical function, without THA.	No serious complication were noted.
Tabatabaee [85]<br>Jarthroplasty<br>2015	Level I<br>Randomized controlled clinical trial	NR/14<br><br>NR/14	26.8<br><br>31	ARCO I: 5<br>ARCO II: 16<br>ARCO III: 7	Steroid: 19<br>Idiopathic: 9	CD alone<br><br>CD + BMC implantation	24 m	3<br><br>0	BMC injection with CD could be an effective therapy for the early stages of AVN, with score improvement.	No serious complication were noted in both the clinical groups.
Daltro [14]<br>Stem Cell Res Ther<br>2015	Level IV<br>Case series	89/89	33<br>(18-55)	Ficat 0: 20<br>Ficat I: 31<br>Ficat IIA: 16<br>Ficat IIB: 22	Sickle Cell Disease	Injection of BMC through a percutaneous approach in the center of the osteonecrotic area	37.3 m<br>(12-60 m)	0	Injection of BMC provides significant improvement of HHS score in all AVN stages, as well as reduces pain, ameliorates quality of daily activities, and prevents the progression of the diseases.	No complications were observed during or after the treatment.
Mao [62]<br>J BoneMiner Res<br>2015	Level I<br>Randomized controlled clinical trial	25/41<br><br>30/48	36.1<br><br>34.6	ARCO I: 18<br>ARCO II: 52<br>ARCO IIIA: 19	Steroid: 31<br>Alcohol: 32<br>Idiopathic: 26	Biomechanical support to the subchondral bone (porous tantalum rod implantation)<br><br>Biomechanical support + Intra-arterially injection of peripheral blood MSC mobilized by G-CSF	36 m	9<br><br>3	Combination treatment provides superior results regarding clinical outcome such as pain, function, activity, and motion compared to biomechanical support alone.	No complication was observed.
Zhao [106]<br>Biomed Res Int<br>2015	Level IV<br>Case series	24/31	33.21<br>(23–45)	ARCO IIIc: 19<br>ARCO IV: 12	Idiopathic: 4<br>Steroid: 14<br>Alcohol: 4<br>Trauma: 2	Cultured bone-marrow derived MSC transplantation associated with porous tantalum rod implantation combined with vascularized iliac grafting	64.4 m<br>(26–78m)	5	The treatment provides improvements of HHS score in all AVN stages.	No complication was observed.
Wang [92]<br>Eur Orthop Surg Traumatol<br>2014	Level IV<br>Case series	15/20	35<br>(23-58)	ARCO IIB: 10<br>ARCO IIC: 6<br>ARCO IIIA: 3<br>ARCO IIIB: 1	Steroid: 4<br>Alcohol: 4<br>Idiopathic: 12	BMAC mixed with cortical and cancellous bone (harvested from the ipsilateral crest) were impacted into the excavated area	24 m<br>(9-36 m)	NR	The treatment provides an overall success of 80 %, their HHS had significant improvement and this new method was best for early-stage small lesions.	No complications were observed.

Cai [8]<br>Transplant Proc<br>2014	Level IV<br>Case series	30/49	41.6<br>(19-63)	ARCO II: 24<br>ARCO III: 25	Steroid: 12<br>Alcohol: 9<br>Idiopathic: 9	Intra−arterially injection of allogeneic human umbilical cord-derived MSCs and BMC	16.9 m<br>(12-21)	0	The treatment provide significant therapeutic effects in AVN, without conversions to THA.	No complication was observed.
Sun [84]<br>PLoS One<br>2014	Level III<br>Retrospective comparative study	39	30.7	ARCO IIb: 6<br>ARCO IIc: 18<br>ARCO IIIa: 19	A majority of the patients had a history of SARS and were treated with high-dose corticosteroids.	Standard background therapy (impacted bone grafting)	6.1 y<br>(5-7.7 y)	NR	The use of rhBMP-2 could be useful to improves the speed and quality of the bone repair but this study lacks of statistical difference.	1 ectopic ossification<br>2 lateral femoral cutaneous nerve lesion
		33	31.1	ARCO IIb: 4<br>ARCO IIc: 21<br>ARCO IIIa: 11		Standard background therapy + rhBMP-2		NR		2 ectopic ossification<br>2 lateral femoral cutaneous nerve lesion
Aoyama [4]<br>Tissue Eng Part B Rev<br>2014	Level IV<br>Case series	10/10	31.7<br>(20-48)	ARCO 3A: 6<br>ARCO 3B: 4<br>Steinberg 3: 6<br>Steinberg 4: 4	Steroid: 4<br>Idiopathic: 6	Cultured bone-marrow derived MSC + vascularized iliac bone graft	24 m	0	This procedure is safe and no conversion to THA was registered, with clinical improvement and bone regeneration.	Frequent complications: increase in creatine phosphokinase and C-reactive protein, anemia, hip pain, decrease in albumin and total protein, complications at the wounded area, wound pain, and fever.
Novais [67]<br>J Pediatr Orthop<br>2014	Level IV<br>Retrospective case series	11/14	12.7<br>(9.7-18)	Steinberg 1B: 1<br>Steinberg 1C: 3<br>Steinberg 2C: 1<br>Steinberg 3C: 1<br>Steinberg 4A: 1<br>Steinberg 4B: 2<br>Steinberg 4C: 5	Sickle Cell Disease	Multiple epiphyseal drilling and BMC implantation	25 m<br>(12-47 m)	2	This treatment provides statistically significant improvement in hip pain and motion.	No complication was observed.
Ma [60]<br>Stem Cell Res Ther<br>2014	Level I<br>Randomized controlled clinical trial	18/24	34.8	Ficat I: 7<br>Ficat II: 32<br>Ficat III: 10	Steroid: 26<br>Alcohol: 7<br>Idiopathic: 12	CD + autologous bone graft	24 m	4	Implantation of the autologous BMC grafting combined with CD is effective to prevent further progression for the early stages of AVN.	No complication was observed.
		21/25	35.6			CD + autologous bone graft with BMC		2		
Chotivichit [11]<br>J Med Assoc Thai<br>2014	Level IV<br>Retrospective case series	32/34	31.9<br>(14-54)	Ficat II: 21<br>Ficat III: 13	Steroid: 26<br>SLE: 9<br>Idiopathic: 5	CD + BMAC	24.6 m<br>stage II<br>27.8 m<br>stage III	NR	CD + injection of BMAC provides only fair results in stage II and III, with 4 THA in stage II and 5 THA in stage III.	No complication was observed.

Calori [9]<br>Injury<br>2014	Level IV<br>Retrospective case series	38/40	46.3<br>(21-73)	Ficat I: 7<br>Ficat II: 25<br>Ficat III: 8	Post-traumatic: 4<br>Steroid: 7<br>Idiopathic: 27	CD and implantation of bone marrow aspirate + growth factors (rhBMP-7) + scaffold of xenograft bone substitute was inserted inside the tunnel of the femur	36 m	5	CD + recombinant morphogenetic proteins + bone marrow aspirate + xenograft bone substitute decrease the incidence of fractural stage non-traumatic osteonecrosis and the progression of AVN and pain.	4 cases of calcification in the soft tissue near the surgery access.<br>1 subtrochanteric fracture of the femur 1 week after CD.
Martin [64]<br>Croat Med J<br>2013	Level IV<br>Retrospective case series	49/73	43	Ficat I: 57<br>Ficat II: 16	Steroid: 44<br>Alcohol: 10<br>Idiopathic: 9<br>Other: 10	CD + adult BMC + PRP are injected into the area of osteonecrosis	17 m	16	This treatment provides a significant pain relief in 86% of patients, satisfactory results in patients with early stage AVN and can lead to complete resolution of the necrotic lesion.	2 post-operative trochanteric bursitis at immediate f-up.
Mao [61]<br>Bone<br>2013	Level IV<br>Case series	62/78	36.3<br>(22-54)	Ficat I: 16<br>Ficat II: 52<br>Ficat III: 10	Steroid: 30<br>Alcohol: 27<br>Idiopathic: 9<br>Trauma: 12	CD (grafting)+Intra-arterial BMC via medial circumflex femoral artery	4.8 y<br>(1-5)	6	The intra-arterial delivery of autologous BMC provides relief of symptoms, improves hip function and delays the progression of the disease.	No complication was observed.
Lim [54]<br>Exp Mol Med<br>2013	Level III<br>Retrospective comparative study	NR/31	34.4	Ficat IIa: 56<br>Ficat IIb: 47<br>Ficat III: 57	Steroid: 54<br>Alcohol: 24<br>Idiopathic: 25<br>Other: 4	CD, curettage and a bone graft	87 m<br>(8-134)	11	Comparable outcomes between multiple drilling + BMC implantation and CD techniques, with a failure rate of 43% and 45.2%, respectively, after 5 years.	No complications were observed.
		NR/159	36.3			Multiple drilling and BMC		47		
Rastogi [74]<br>Musculoskelet Surg<br>2013	Level I<br>Randomized controlled clinical trial	20/30	33.0	NR	Steroid: 18<br>Alcohol: 8<br>Idiopathic: 26<br>Smoking: 8	CD and unprocessed bone marrow injection	24 m	3	Control and treatment group shows significant differences when compared with pre-operative scores, without statistically significant inter-group differences in clinical scores. 3 THA in the group treated with CD and unprocessed bone marrow injection.	No complications were noted in both groups.
		20/30	34.7			CD + BMC implantation		3		

Liu [55]<br>Arch Orthop Trauma Surg<br>2013	Level III<br>Retrospective comparative study	NR/27	38.1	ARCO IIb: 25<br>ARCO IIc: 30	Steroid: 19<br>Alcohol: 29<br>Idiopathic: 7	CD with implantation of porous nano-hydroxylapatite/polyamide 66 composite bone filler	24.9	5	CD + BMC treatment provides better outcomes respect to CD alone in decrease hip pain, improve hip function, preventing collapse of the femoral head, with 4 failures, compared to 5 in control group.	No complications were observed.
		NR/28	38.0			CD with implantation of BMC with porous nano-hydroxylapatite/ polyamide 66 composite bone filler	26.7	4		
Aarvold [1]<br>Surgeon<br>2013	Level IV<br>Case series	4/5	36.2	Ficat II: 5	Steroid: 3<br>Alcohol: 1<br>Idiopathic: 1	CD + impaction of BMC/milled allograft (obtained from frozen femoral heads) construct	44 m	2	This treatment shows the potential of BMC/allograft constructs for the treatment of early stage AVN.	No complications were observed.
Kang [44]<br>Yonsei Med J<br>2013	Level IV<br>Case series	52/61	43.8<br>(19-66)	ARCO I: 5<br>ARCO II: 35<br>ARCO III: 18<br>ARCO IV: 3	Steroid: 6<br>Alcohol: 16<br>Idiopathic: 22<br>Trauma: 6	CD combined with auto iliac bone graft and implantation of BMC	68 m<br>(60-88)	NR	Overall clinical results of our procedure were not satisfactory, with 26 hip having bad or failed clinical results, particularly in patients with large lesions	No complications were observed.
Pak [68]<br>Pain Physician<br>2012	Level IV<br>Case report	2	34 and 39	NR	NR	Injection of adipose tissue-derived stem cells with hyaluronic acid, PRP and CaCl^2 to activate PRP. Then PRP + Cacl^2 injection every 4 weeks	12 m	NR	The injection of stem cell mixture demonstrate the presence of newly regenerated tissue in 2 severely necrotic femoral heads.	No complications were observed.
Sen [80]<br>J Arthroplasty<br>2012	Level I<br>Randomized controlled clinical trial	NR/25	NR	ARCO I, II	Steroid: 14<br>Alcohol: 6<br>Idiopathic: 1<br>Pregnancy: 1<br>Cushing disease: 1<br>Trauma: 17	CD alone	24 m	6	BMC instillation can result in better clinical outcome and hip survival, with only 1 THA in treatment group vs 6 in control group.	No complications were observed.
		NR/26				CD + BMC implantation		1		
Civinini [12]<br>Int Orthop<br>2012	Level IV<br>Case series	31/37	43.9<br>(24–56)	Steinberg Ic: 3<br>Steinberg IIa: 7<br>Steinberg IIb: 11<br>Steinberg IIc: 9<br>Steinberg IIIa: 7	Steroid: 14<br>Bone marrow transplantation: 4<br>Alcohol: 10<br>SLE: 1<br>Idiopathic: 6	CD + injection of BMC + new composite injectable bone substitute (PRO-DENSE®)	20.6 m<br>(12-32)	3	Significant improvement of function and symptoms. Radiological success in patient with stage I.<br>This technique was best for early-stage lesions.	No complications related to the procedure were seen during or after the operation.

Zhao [105]<br>Bone<br>2012	Level I<br>Randomized controlled clinical trial	NR/44	33.1<br>(18-55)	ARCO IC: 5<br>ARCO IIA: 30<br>ARCO IIB: 46<br>ARCO IIC: 23	Steroid: 24<br>Alcohol: 19<br>Idiopathic: 30<br>Trauma: 20<br>Caisson disease: 11	CD alone	60 m	10	Ex vivo expansion of bone-marrow derived MSC and implantation provides significantly improvement of pain and other joint symptoms and delay or avoid the progression of osteonecrosis and total hip replacement.	No complications were observed.
		NR/53				CD with cultured bone-marrow derived MSC		2		
Yoshioka [101]<br>Int Orthop<br>2011	Level IV<br>Case series	6/9	31.5<br>(16–52)	JOA 1: 2<br>JOA 2: 4<br>JOA 3A: 1<br>JOA 3B: 2	Corticosteroid-induced AVN in SLE	CD + BMC implantation	41 m<br>(37-53)	1	BMC treatment provides a significant improvement of pain and function.	No complications were observed.
Gangji [20]<br>Bone<br>2011	Level I<br>Double blinded controlled clinical trial	NR/11	45.7	ARCO I: 4<br>ARCO II: 20	Steroid: 20<br>Alcohol: 2<br>Idiopathic: 2	CD alone	60 m	3	BMC implantation in the necrotic lesion provides better results in early AVN and delay its progression, reduces pain and decreases the volume of necrotic lesion.	No complications were observed.
		NR/13	42.2			CD + BMC implantation		2		
Yamasaki [95]<br>J Bone Joint Surg<br>2010	Level III<br>Retrospective comparative study	NR/9	49	JOA 1: 2<br>JOA 2: 34<br>JOA 3A: 3	Steroid: 24<br>Alcohol: 10<br>Idiopathic: 5	Implantation of cell-free IP-CHA scaffold into the site of osteonecrosis	31 m	3	BMC treatment provide a progression of bone repair, accelerates revascularisation along the transition zone.<br>This study described the apparent effectiveness of implantation of BMC with IP-CHA.	No intra- or post-operative complications were observed in either group.
		NR/30	41			Transplantation of BMC-seeded IP-CHA	29 m	1		
Wang [91]<br>Arch Orthop Trauma Surg<br>2010	Level IV<br>Case series	45/59	37.5<br>(16–56)	ARCO I: 2<br>ARCO IIA: 7<br>ARCO IIB: 13<br>ARCO IIC: 28<br>ARCO IIIA: 9	Steroid: 29<br>Alcohol: 22<br>Idiopathic: 8	CD + BMC implantation	27.6 m<br>(12-40)	7	CD + BMC significantly improved the joint pain, delayed the joint replacement and it is indicated for the treatment of stage I and II of AVN.	No complications were seen during or after the operation.
Hernigou [37]<br>Indian J Orthop<br>2009	Level IV<br>Case series	342/534	39<br>(16–61)	Steinberg I-II	Steroid: 102<br>Alcohol: 150<br>SCD: 282	CD + BMC implantation	13 y<br>(8-18)	NR	This treatment provide a complete resolution of AVN in 69 patients.	No complications were observed.
Hendrich [32]<br>Orthop Rev<br>2009	Level IV<br>Case series	37	NR	NR	NR	CD + BMC implantation	14 m<br>(2-24)	1	Good results in AVN and safety were proved.	No specific complication.
Seyler [81]<br>Clin Orthop Relat Res<br>2008	Level IV<br>Case series	33/39	35<br>(18-52)	Ficat II: 22<br>Ficat III: 17	Steroid: 12<br>Alcohol: 8<br>SLE: 7<br>Tobacco: 4<br>Hepatitis C: 3<br>HIV: 2<br>Other: 3	CD with nonvascularized bone grafting procedures + OP-1 (BMP 7)	36 m<br>(24-50)	13	Significant improvement of mean HHS. This is an effective and safe procedure.	No complications were observed.

Yamasaki [96] Med Sci Monit 2008	Level IV Case report	2/4	Case 1: 18 Case 2: 40	ARCO II: 2 ARCO III: 2	Alcool: 2 Idiopathic: 2	Transplantation of BMC into the affected area of one hip (left hip) using IP-CHA in two patients, while the other hip was simultaneously treated with transtrochanteric rotational osteotomy	22 m	0	Transplantation of BMC may provide a beneficial treatment for bone repair in the condition of ON.	No complications were observed.
Kawate [46] Artif Organs 2006	Level IV Case report	3/4	28 (25–30)	Steinberg IVA: 1 Steinberg IVC: 3	Steroid: 4	Cultured bone-marrow derived MSC transplantation with b-TCP ceramics	34 m (27-48)	0	The MSCs/b-TCP transplantation provides a significant sclerotic change at the beginning. After 1 year it could be noted a revascularization, probably derived from the vascularized fibula. This procedure is not indicated for cases with severe preoperative collapse.	No complications were observed.
Lieberman [53] Clin Orthop Relat Res 2004	Level IV Case series	15/17	47 (36-62)	Ficat IIa: 15 Ficat IIb: 1 Ficat III: 1	Steroid: 14 Alcohol: 4	CD + hBMP	53 m (26-94)	3	CD + Human bone morphogenetic protein provide relief of pain, delay the radiographic progression.	No complications were observed.
Gangji [21] J Bone Joint Surg 2004	Level II Prospective comparative study	NR/8	48.8	ARCO I: 2 ARCO II: 16	Steroid: 14 Alcohol: 2 Idiopathic: 2	CD alone	24 m	NR	CD+BMC provide significant decrease in the level of pain, and other joint symptoms. The volume of necrotic lesions significantly improve only in treatment group.	No complications were observed.
		NR/10	40.9			CD + BMC implantation		0		
Hernigou [35] Clin Orthop Relat Res 2002	Level IV Case series	116/189	31 (16-61)	Steinberg I: 59 Steinberg II: 86 Steinberg III: 12 Steinberg IV: 32	Steroid: 31 Alcohol: 56 Idiopathic: 10 SCD: 64 Organ transplantation: 21 Others: 7	CD + BMC implantation	7 y (5-11)	34	Patients with hips in stage III and IV preoperatively had a poor results and progressed in stages.	No specific complication.

Characteristics of included studies.

Adipose-derived mesenchymal stem cell (ADMSC); Angioconductive bioceramic rod (ABR); Association Research Circulation Osseous (ARCO); Autologous bone marrow buffy coat (BBC); Autologous bone marrow derived osteoblasts (ABMDO); Autologous lipoaspirate cells (lpcs); Avascular necrosis (AVN); Body mass index (BMI); Bone graft (BG); Bone marrow aspirate concentrate (BMAC); Bone marrow mesenchymal stromal cells (BM-mscs); Bone marrow mononuclear cells (bmmcs);

Bone morphogenetic protein (BMP); Concentrated autologous bone marrow aspirate transplantation (CABMAT); Core decompression (CD); Extracorporeal shock wave therapy (ESWT); Follow-up (F-up); Granulocyte-colony stimulating factor (G-CSF); Harris hip score (HHS); Human Bone Marrow Mesenchymal Stem Cell (hbmscs); Human Mesenchymal Stem Cells (HMSC); Human umbilical cord-derived mscs (huc-mscs); Japanese International Committee (JIC); Japanese Orthopaedic Association (JOA); Low-intensity pulsed ultrasound (LIPUS); Lupus erythematosus (SLE); Mesenchymal stromal cells (mscs); Months (m); Not reported (NR); Peripheral blood stem cells (pbscs); Platelet-rich plasma (PRP); Randomized controlled trials (rcts); Recombinant human bone morphogenetic protein (rhbmp); Recombinant human fibroblast growth factor-2 (rhfgf-2); Sickle cell disease (SCD); Total hip arthroplasty (THA); Tricalcium phosphate (TCP); Years (y).

References

1. Aarvold A, Smith JO, Tayton ER, Jones AMH, Dawson JI, Lanham S, Briscoe A, Dunlop DG, Oreffo ROC (2013) A tissue engineering strategy for the treatment of avascular necrosis of the femoral head. Surgeon 11(6):319–325

2. Aggarwal AK, Poornalingam K, Jain A, Prakash M (2021) Combining Platelet-Rich Plasma Instillation With Core Decompression Improves Functional Outcome and Delays Progression in Early-Stage Avascular Necrosis of Femoral Head: a 4.5- to 6-Year Prospective Randomized Comparative Study. The Journal of Arthroplasty 36(1):54–61

3. Aoyama T, Fujita Y, Madoba K, Nankaku M, Yamada M, Tomita M, Goto K, Ikeguchi R, Kakinoki R, Matsuda S, Nakamura T, Toguchida J (2015) Rehabilitation Program After Mesenchymal Stromal Cell Transplantation Augmented by Vascularized Bone Grafts for Idiopathic Osteonecrosis of the Femoral Head: A Preliminary Study. Archives of Physical Medicine and Rehabilitation 96(3):532–539

4. Aoyama T, Goto K, Kakinoki R, Ikeguchi R, Ueda M, Kasai Y, Maekawa T, Tada H, Teramukai S, Nakamura T, Toguchida J (2014) An Exploratory Clinical Trial for Idiopathic Osteonecrosis of Femoral Head by Cultured Autologous Multipotent Mesenchymal Stromal Cells Augmented with Vascularized Bone Grafts. Tissue Engineering Part B: Reviews 20(4):233–242

5. Baghdadi S, Chern I, Hanstein R, Mehraban Alvandi L, Fornari E (2023) Femoral Head Core Decompression and Bone Marrow Concentrate Injection in Pediatric Sickle-cell Related Avascular Necrosis. J Pediatr Orthop 43(6):e433–e439

6. Blanco JF, Garcia-Garcia FJ, Villarón EM, da Casa C, Fidalgo H, López-Parra M, Santos JA, Sánchez-Guijo F (2023) Long-Term Results of a Phase I/II Clinical Trial of Autologous Mesenchymal Stem Cell Therapy for Femoral Head Osteonecrosis. J Clin Med 12(6):2117

7. Boontanapibul K, Huddleston JI, Amanatullah DF, Maloney WJ, Goodman SB (2021) Modified Kerboul Angle Predicts Outcome of Core Decompression With or Without Additional Cell Therapy. The Journal of Arthroplasty 36(6):1879–1886

8. Cai J, Wu Z, Huang L, Chen J, Wu C, Wang S, Deng Z, Wu W, Luo F, Tan J (2014) Cotransplantation of Bone Marrow Mononuclear Cells and Umbilical Cord Mesenchymal Stem Cells in Avascular Necrosis of the Femoral Head. Transplantation Proceedings 46(1):151–155

9. Calori GM, Mazza E, Colombo M, Mazzola S, Mineo GV, Giannoudis PV (2014) Treatment of AVN using the induction chamber technique and a biological-based approach: Indications and clinical results. Injury 45(2):369–373

10. Chen C, Qu Z, Yin X, Shang C, Ao Q, Gu Y, Liu Y (2016) Efficacy of umbilical cord-derived mesenchymal stem cell-based therapy for osteonecrosis of the femoral head: A three-year follow-up study. Molecular Medicine Reports 14(5):4209–4215

11. Chotivichit A, Korwutthikulrangsri E, Pornrattanamaneewong C, Achawakulthep C (2014) Core decompression with bone marrow injection for the treatment of femoral head osteonecrosis. J Med Assoc Thai 97 Suppl 9:S139-143

12. Civinini R, De Biase P, Carulli C, Matassi F, Nistri L, Capanna R, Innocenti M (2012) The use of an injectable calcium sulphate/calcium phosphate bioceramic in the treatment of osteonecrosis of the femoral head. International Orthopaedics (SICOT) 36(8):1583–1588

13. Cruz-Pardos A, Garcia-Rey E, Ortega-Chamarro JA, Duran-Manrique D, Gomez-Barrena E (2016) Mid-Term Comparative Outcomes of Autologous Bone-Marrow Concentration to Treat Osteonecrosis of the Femoral Head in Standard Practice. HIP International 26(5):432–437

14. Daltro GC, Fortuna V, de Souza ES, Salles MM, Carreira AC, Meyer R, Freire SM, Borojevic R (2015) Efficacy of autologous stem cell-based therapy for osteonecrosis of the femoral head in sickle cell disease: a five-year follow-up study. Stem Cell Research & Therapy 6(1):110

15. D'Ambrosi R, Biancardi E, Massari G, Ragone V, Facchini RM (2018) Survival Analysis after Core Decompression in Association with Platelet-Rich Plasma, Mesenchymal Stem Cells, and Synthetic Bone Graft in Patients with Osteonecrosis of the Femoral Head. Joints 6(1):16–22

16. Davulcu CD, Karaismailoğlu B, OzsahiN MK, Davutluoglu E, Akbaba D, Terzi E, Ünlü MC (2023) Autologous bone plug-sliding with core decompression and bone marrow aspirate concentrate application: a joint-preserving surgical technique for corticosteroid-induced osteonecrosis of femoral head. Acta Orthop Belg 89(4):603–608

17. Döring M, Kluba T, Cabanillas Stanchi KM, Kahle P, Lenglinger K, Tsiflikas I, Treuner C, Vaegler M, Mezger M, Erbacher A, Schumm M, Lang P, Handgretinger R, Müller I (2020) Longtime Outcome After Intraosseous Application of Autologous Mesenchymal Stromal Cells in Pediatric Patients and Young Adults with Avascular Necrosis After Steroid or Chemotherapy. Stem Cells and Development 29(13):811–822

18. Einhorn TA, Anoushiravani AA, Chen KK, Elbuluk A, Tsismenakis T, Lespasio M, Iorio R (2017) Treatment of stage I and II osteonecrosis of the femoral head with core decompression and bone marrow aspirate concentrate injection—A 2-year follow-up study. Seminars in Arthroplasty 28(4):239–245

19. Emadedin M, Karimi S, Karimi A, Labibzadeh N, Niknejadi M, Baharvand H, Aghdami N (2019) Autologous bone marrow-derived CD133 cells with core decompression as a novel treatment method for femoral head osteonecrosis: a pilot study. Cytotherapy 21(1):107–112

20. Gangji V, De Maertelaer V, Hauzeur J-P (2011) Autologous bone marrow cell implantation in the treatment of non-traumatic osteonecrosis of the femoral head: Five year follow-up of a prospective controlled study. Bone 49(5):1005–1009

21. Gangji V, Hauzeur J-P, Matos C, De Maertelaer V, Toungouz M, Lambermont M (2004) Treatment of osteonecrosis of the femoral head with implantation of autologous bone-marrow cells. A pilot study. J Bone Joint Surg Am 86(6):1153–1160

22. Gao F, Sun W, Guo W, Wang B, Cheng L, Li Z (2016) Combined with Bone Marrow-Derived Cells and rhBMP-2 for Osteonecrosis after Femoral Neck Fractures in Children and Adolescents: A case series. Sci Rep 6:30730

23. Gao H, Zhang G, Wang J, Zhao F, Zhang Y, Wang W, You Y (2015) Clinical Effects of Novel Nanoscaled Core Decompression Rods Combined with Umbilical Cord Mesenchymal Stem Cells on the Treatment of Early Osteonecrosis of the Femoral Head. Journal of Nanomaterials 2015:e902836

24. Gianakos AL, Moya-Angeler J, Duggal S, Zambrana L, Fields KG, Mintz DN, Cornell CN, Lane JM (2016) The Efficacy of Bisphosphonates with Core Decompression and Mesenchymal Stem Cells Compared with Bisphosphonates Alone in the Treatment of Osteonecrosis of the Hip: a Retrospective Study. HSS J 12(2):137–144

25. Gómez-Barrena E, Padilla-Eguiluz N-G, López-Marfil M, Ruiz de la Reina R (2022) Volume and location of bone regeneration after autologous expanded mesenchymal stromal cells in hip osteonecrosis. Bone Joint Res 11(12):881–889

26. Gómez-Barrena E, Padilla-Eguiluz NG, Rosset P, Hernigou P, Baldini N, Ciapetti G, Gonzalo-Daganzo RM, Avendaño-Solá C, Rouard H, Giordano R, Dominici M, Schrezenmeier H, Layrolle P, On Behalf Of The Reborne Consortium null (2021) Osteonecrosis of the Femoral Head Safely Healed with Autologous, Expanded, Bone Marrow-Derived Mesenchymal Stromal Cells in a Multicentric Trial with Minimum 5 Years Follow-Up. J Clin Med 10(3):508

27. Goto K, Aoyama T, Toguchida J, Kuroda Y, Kawai T, Okuzu Y, Matsuda S (2021) Ten-year results of mesenchymal stromal cell transplantation augmented with vascularised bone grafts for advanced osteonecrosis of the femoral head. J Orthop 26:67–71

28. Grassi M, Salari P, Massetti D, Papalia GF, Gigante A (2020) Treatment of avascular osteonecrosis of femoral head by core decompression and platelet-rich plasma: a prospective not controlled study. International Orthopaedics (SICOT) 44(7):1287–1294

29. H. Majeed G, Sh Al-Edanni M, Abbood LJ, Al-Mukhtar SA (2021) Early Stages Non-Traumatic Avascular Necrosis of Femoral Head Treated by Core Decompression With and Without Platelets Rich Plasma Injection: A Comparative Study. ACTADOI: 10.18502/acta.v58i11.5144

30. Hauzeur J-P, De Maertelaer V, Baudoux E, Malaise M, Beguin Y, Gangji V (2018) Inefficacy of autologous bone marrow concentrate in stage three osteonecrosis: a randomized controlled double-blind trial. Int Orthop 42(7):1429–1435

31. Hauzeur J-P, Lechanteur C, Baudoux E, De Maertelaer V, Pather S, Katz R, Malaise M, Ino J, Beguin Y (2020) Did Osteoblastic Cell Therapy Improve the Prognosis of Pre-fracture Osteonecrosis of the Femoral Head? A Randomized, Controlled Trial. Clin Orthop Relat Res 478(6):1307–1315

32. Hendrich C, Engelmaier F, Waertel G, Krebs R, Jäger M (2009) Safety of autologous bone marrow aspiration concentrate transplantation: initial experiences in 101 patients. Orthopedic Reviews 1(2):e32–e32

33. Hernandez A, Nuñez JH, Sallent A, Gargallo-Margarit A, Gallardo-Calero I, Barro V (2020) Core Decompression Combined with Implantation of Autologous Bone Marrow Concentrate with Tricalcium Phosphate Does Not Prevent Radiographic Progression in Early Stage Osteonecrosis of the Hip. Clin Orthop Surg 12(2):151–157

34. Hernigou J, Housset V, Dubory A, Flouzat Lachaniette CH, Rouard H, Hernigou P (2021) Cell therapy for post-traumatic hip osteonecrosis in young patients. Morphologie 105(349):127–133

35. Hernigou P, Beaujean F (2002) Treatment of osteonecrosis with autologous bone marrow grafting. Clin Orthop Relat ResDOI: 10.1097/00003086-200212000-00003

36. Hernigou P, Dubory A, Homma Y, Guissou I, Flouzat Lachaniette CH, Chevallier N, Rouard H (2018) Cell therapy versus simultaneous contralateral decompression in symptomatic corticosteroid osteonecrosis: a thirty year follow-up prospective randomized study of one hundred and twenty five adult patients. International Orthopaedics (SICOT) 42(7):1639–1649

37. Hernigou P, Poignard A, Zilber S, Rouard H (2009) Cell therapy of hip osteonecrosis with autologous bone marrow grafting. Indian J Orthop 43(1):40–45

38. Hernigou P, Thiebaut B, Housset V, Bastard C, Homma Y, Chaib Y, Flouzat Lachaniette CH (2018) Stem cell therapy in bilateral osteonecrosis: computer-assisted surgery versus conventional fluoroscopic technique on the contralateral side. International Orthopaedics (SICOT) 42(7):1593–1598

39. Hong Z, Zhang Y, Chen J, Bi Q (2023) Adipose-derived stromal vascular fraction injection following core decompression and biochemistry artificial bone graft implantation in osteonecrosis of the femoral head. International Orthopaedics (SICOT) 47(6):1481–1486

40. Hoogervorst P, Campbell JC, Scholz N, Cheng EY (2022) Core Decompression and Bone Marrow Aspiration Concentrate Grafting for Osteonecrosis of the Femoral Head. J Bone Joint Surg Am 104(Suppl 2):54–60

41. Houdek MT, Wyles CC, Smith J-RH, Terzic A, Behfar A, Sierra RJ (2021) Hip decompression combined with bone marrow concentrate and platelet-rich plasma for corticosteroid-induced osteonecrosis of the femoral head. Bone Jt Open 2(11):926–931

42. Hyodo K, Yoshioka T, Sugaya H, Akaogi H, Aoto K, Wada H, Shimizu Y, Yamazaki M, Mishima H (2017) Predicting Risk Factors of Total Hip Arthroplasty Conversion after Concentrated Autologous Bone Marrow Aspirate Transplantation for the Treatment of Idiopathic Osteonecrosis of the Femoral Head: A Retrospective Review of 213 Hips at a Mean Follow-up of 5 Years. The Journal of Hip Surgery 01(01):007–013

43. Jayankura M, Thomas T, Seefried L, Dubrana F, Günther K-P, Rondia J, Davis ET, Winnock de Grave P, Carron P, Gangji V, Vande Berg B, Godeaux O, Sonnet W (2023) Does Adjunction of Autologous Osteoblastic Cells Improve the Results of Core Decompression in Early-stage Femoral Head Osteonecrosis? A Double-blind, Randomized Trial. Clinical Orthopaedics and Related Research® 481(8):1527

44. Kang JS, Moon KH, Kim B-S, Kwon DG, Shin SH, Shin BK, Ryu D-J (2013) Clinical Results of Auto-Iliac Cancellous Bone Grafts Combined with Implantation of Autologous Bone Marrow Cells for Osteonecrosis of the Femoral Head: A Minimum 5-Year Follow-Up. Yonsei Medical Journal 54(2):510–515

45. Kang JS, Suh YJ, Moon KH, Park JS, Roh TH, Park MH, Ryu DJ (2018) Clinical efficiency of bone marrow mesenchymal stem cell implantation for osteonecrosis of the femoral head: a matched pair control study with simple core decompression. Stem Cell Res Ther 9(1):274

46. Kawate K, Yajima H, Ohgushi H, Kotobuki N, Sugimoto K, Ohmura T, Kobata Y, Shigematsu K, Kawamura K, Tamai K, Takakura Y (2006) Tissue-engineered Approach for the Treatment of Steroid-induced Osteonecrosis of the Femoral Head: Transplantation of Autologous Mesenchymal Stem Cells Cultured With Beta-Tricalcium Phosphate Ceramics and Free Vascularized Fibula. Artificial Organs 30(12):960–962

47. Khan M, Abbas K, Ayling E, Waqas Ilyas M, Dunlop D (2021) Autologous stem cell implantation with core decompression for avascular necrosis of the femoral head using a new device. annals 103(7):508–513

48. Kuroda Y, Asada R, So K, Yonezawa A, Nankaku M, Mukai K, Ito-Ihara T, Tada H, Yamamoto M, Murayama T, Morita S, Tabata Y, Yokode M, Shimizu A, Matsuda S, Akiyama H (2016) A pilot study of regenerative therapy using controlled release of recombinant human fibroblast growth factor for patients with pre-collapse osteonecrosis of the femoral head. International Orthopaedics (SICOT) 40(8):1747–1754

49. Kuroda Y, Tanaka T, Miyagawa T, Hamada H, Abe H, Ito-Ihara T, Asada R, Fujimoto Y, Takahashi D, Tetsunaga T, Kaneuji A, Takagi M, Inaba Y, Morita S, Sugano N, Tanaka S, Matsuda S, Akiyama H, TRION trial collaborators: (2021) Recombinant human FGF-2 for the treatment of early-stage osteonecrosis of the femoral head: TRION, a single-arm, multicenter, Phase II trial. Regen Med 16(6):535–548

50. Li M, Ma Y, Fu G, Zhang R, Li Q, Deng Z, Zheng M, Zheng Q (2020) 10-year follow-up results of the prospective, double-blinded, randomized, controlled study on autologous bone marrow buffy coat grafting combined with core decompression in patients with avascular necrosis of the femoral head. Stem Cell Res Ther 11(1):287

51. Li Q, Liao W, Fu G, Liao J, Zhang R, Li M, Yang Y, Ma Y, Zheng M, Zheng Q (2021) Combining autologous bone marrow buffy coat and angioconductive bioceramic rod grafting with advanced core decompression improves short-term outcomes in early avascular necrosis of the femoral head: a prospective, randomized, comparative study. Stem Cell Res Ther 12(1):354

52. Liang D, Pei J, Zhang X, Chen X (2023) Clinical outcomes of autologous platelet-rich plasma and bone marrow mononuclear cells grafting combined with core decompression for Association Research Circulation Osseous II–IIIA stage non-traumatic osteonecrosis of the femoral head. International Orthopaedics (SICOT) 47(9):2181–2188

53. Lieberman JR, Conduah A, Urist MR (2004) Treatment of osteonecrosis of the femoral head with core decompression and human bone morphogenetic protein. Clin

Orthop Relat ResDOI: 10.1097/01.blo.0000150312.53937.6f

54. Lim YW, Kim YS, Lee JW, Kwon SY (2013) Stem cell implantation for osteonecrosis of the femoral head. Exp Mol Med 45(11):e61–e61

55. Liu CC, Solderer A, Heumann C, Attin T, Schmidlin PR (2021) Tricalcium phosphate (-containing) biomaterials in the treatment of periodontal infra-bony defects: A systematic review and meta-analysis. Journal of Dentistry 114:103812

56. Liu L, Gao F, Sun W, Wang Y, Zhang Q, Wang B, Cheng L, Li Z (2018) Investigating clinical failure of core decompression with autologous bone marrow mononuclear cells grafting for the treatment of non-traumatic osteonecrosis of the femoral head. International Orthopaedics (SICOT) 42(7):1575–1583

57. Luan S, Wang S, Lin C, Fan S, Liu C, Ma C, Wu S (2022) Comparisons of Ultrasound-Guided Platelet-Rich Plasma Intra-Articular Injection and Extracorporeal Shock Wave Therapy in Treating ARCO I–III Symptomatic Non-Traumatic Femoral Head Necrosis: A Randomized Controlled Clinical Trial. J Pain Res 15:341–354

58. Lyu J, Ma T, Huang X, Shi J, Huang G, Chen F, Wei Y, Wang S, Xia J, Zhao G, Chen J (2023) Core decompression with β-tri-calcium phosphate grafts in combination with platelet-rich plasma for the treatment of avascular necrosis of femoral head. BMC Musculoskelet Disord 24(1):40

59. Ma H-Y, Ma N, Liu Y-F, Wan Y-Q, Liu G-Q, Liu G-B, Meng H-Y, Li H, Wang X, Li C-B, Peng J (2021) Core Decompression with Local Administration of Zoledronate and Enriched Bone Marrow Mononuclear Cells for Treatment of Non-Traumatic Osteonecrosis of Femoral Head. Orthop Surg 13(6):1843–1852

60. Ma Y, Wang T, Liao J, Gu H, Lin X, Jiang Q, Bulsara MK, Zheng M, Zheng Q (2014) Efficacy of autologous bone marrow buffy coat grafting combined with core decompression in patients with avascular necrosis of femoral head: a prospective, double-blinded, randomized, controlled study. Stem Cell Research & Therapy 5(5):115

61. Mao Q, Jin H, Liao F, Xiao L, Chen D, Tong P (2013) The efficacy of targeted intraarterial delivery of concentrated autologous bone marrow containing mononuclear cells in the treatment of osteonecrosis of the femoral head: A five year follow-up study. Bone 57(2):509–516

62. Mao Q, Wang W, Xu T, Zhang S, Xiao L, Chen D, Jin H, Tong P (2015) Combination Treatment of Biomechanical Support and Targeted Intra-arterial Infusion of Peripheral Blood Stem Cells Mobilized by Granulocyte-Colony Stimulating Factor for the Osteonecrosis of the Femoral Head: A Randomized Controlled Clinical Trial. Journal of Bone and Mineral Research 30(4):647–656

63. Mardones R, Camacho D, Monsalvo F, Zulch N, Jofre C, Minguell JJ (2019) Treatment of osteonecrosis of the femoral head by core decompression and implantation of fully functional ex vivo-expanded bone marrow-derived mesenchymal stem cells: a proof-of-concept study. Stem Cells Cloning 12:11–16

64. Martin JR, Houdek MT, Sierra RJ (2013) Use of concentrated bone marrow aspirate and platelet rich plasma during minimally invasive decompression of the femoral head in the treatment of osteonecrosis. Croat Med J 54(3):219–224

65. Martinot P, Dartus J, Leclerc JT, Putman S, Girard J, Migaud H (2020) Hip survival after plain core decompression alone versus bone morphogenetic protein and/or bone marrow reinjection with core decompression for avascular osteonecrosis of the femoral head: a retrospective case control study in ninety two patients. International Orthopaedics (SICOT) 44(11):2275–2282

66. Mishima H, Sugaya H, Yoshioka T, Aoto K, Wada H, Akaogi H, Ochiai N (2016) The safety and efficacy of combined autologous concentrated bone marrow grafting and low-intensity pulsed ultrasound in the treatment of osteonecrosis of the femoral head. Eur J Orthop Surg Traumatol 26(3):293–298

67. Novais EN, Sankar WN, Wells L, Carry PM, Kim Y-J (2015) Preliminary Results of Multiple Epiphyseal Drilling and Autologous Bone Marrow Implantation for Osteonecrosis of the Femoral Head Secondary to Sickle Cell Disease in Children. Journal of Pediatric Orthopaedics 35(8):810

68. Pak J (2012) Autologous adipose tissue-derived stem cells induce persistent bone-like tissue in osteonecrotic femoral heads. Pain Physician 15(1):75–85

69. Palekar G (2021) Hip Preservation With Autologous Osteoblast Cell-Based Treatment in Osteonecrosis of the Femoral Head. Orthopedics 44(2):e183–e189

70. Pan J, Ding Q, Lv S, Xia B, Jin H, Chen D, Xiao L, Tong P (2020) Prognosis after autologous peripheral blood stem cell transplantation for osteonecrosis of the femoral head in the pre-collapse stage: a retrospective cohort study. Stem Cell Res Ther 11(1):83

71. Pepke W, Kasten P, Beckmann N, Janicki P, Egermann M (2016) Core decompression and autologous bone marrow concentrate for treatment of femoral head osteonecrosis: a randomized prospective study. Orthopedic ReviewsDOI: 10.4081/or.2016.6162

72. Persiani P, De Cristo C, Graci J, Noia G, Gurzì M, Villani C (2015) Stage-related results in treatment of hip osteonecrosis with core-decompression and autologous mesenchymal stem cells. Acta Orthop Belg 81(3):406–412

73. Pilge H, Bittersohl B, Schneppendahl J, Hesper T, Zilkens C, Ruppert M, Krauspe R, Jäger M (2016) Bone Marrow Aspirate Concentrate in Combination With Intravenous Iloprost Increases Bone Healing in Patients With Avascular Necrosis of the Femoral Head: A Matched Pair Analysis. Orthop Rev (Pavia) 8(4):6902

74. Rastogi S, Sankineani SR, Nag HL, Mohanty S, Shivanand G, Marimuthu K, Kumar R, Rijal L (2013) Intralesional autologous mesenchymal stem cells in management of osteonecrosis of femur: a preliminary study. Musculoskelet Surg 97(3):223–228

75. Rocchi M, Del Piccolo N, Mazzotta A, Giavaresi G, Fini M, Facchini F, Stagni C, Dallari D (2020) Core decompression with bone chips allograft in combination with fibrin platelet-rich plasma and concentrated autologous mesenchymal stromal cells, isolated from bone marrow: results for the treatment of avascular necrosis of the femoral head after 2 years minimum follow-up. HIP International 30(2_suppl):3–12

76. de Rojas T, Martínez-Álvarez S, Lerma-Lara S, Díaz MÁ, Madero L, Ramírez M (2018) Outcome of childhood leukaemia survivors and necrosis of the femoral head treated with autologous mesenchymal stem cells. Clin Transl Oncol 20(5):584–590

77. Sadat-Ali M, Al-Omran AS, AlTabash K, Acharya S, Hegazi TM, Al Muhaish MI (2022) The clinical and radiological effectiveness of autologous bone marrow derived osteoblasts (ABMDO) in the management of avascular necrosis of femoral head (ANFH) in sickle cell disease (SCD). J Exp Orthop 9(1):18

78. Sadat-Ali M, Azam MQ, Elshabouri EM, Tantawy AM, Acharya S (2017) Stem Cell Therapy for Avascular Necrosis of Femoral Head in Sickle Cell Disease: Report of 11 Cases and Review of Literature. Int J Stem Cells 10(2):179–183

79. Samy AM (2016) Management of osteonecrosis of the femoral head. IJOO 50(4):359–365

80. Sen RK, Tripathy SK, Aggarwal S, Marwaha N, Sharma RR, Khandelwal N (2012) Early Results of Core Decompression and Autologous Bone Marrow Mononuclear Cells Instillation in Femoral Head Osteonecrosis: A Randomized Control Study. The Journal of Arthroplasty 27(5):679–686

81. Seyler TM, Marker DR, Ulrich SD, Fatscher T, Mont MA (2008) Nonvascularized Bone Grafting Defers Joint Arthroplasty in Hip Osteonecrosis. Clinical Orthopaedics and Related Research® 466(5):1125

82. Shi L, Sun W, Gao F, Cheng L, Li Z (2017) Heterotopic ossification related to the use of recombinant human BMP-2 in osteonecrosis of femoral head. Medicine (Baltimore) 96(27):e7413

83. Sugaya H, Yoshioka T, Tomaru Y, Kumagai H, Yamazaki M, Mishima H (2022) An exploratory clinical trial for concentrated autologous bone marrow aspirate transplantation in the treatment of osteonecrosis of the femoral head. Eur J Orthop Surg TraumatolDOI: 10.1007/s00590-022-03201-6

84. Sun W, Li Z, Gao F, Shi Z, Zhang Q, Guo W (2014) Recombinant Human Bone Morphogenetic Protein-2 in Debridement and Impacted Bone Graft for the Treatment of Femoral Head Osteonecrosis. PLOS ONE 9(6):e100424

85. Tabatabaee RM, Saberi S, Parvizi J, Mortazavi SMJ, Farzan M (2015) Combining Concentrated Autologous Bone Marrow Stem Cells Injection With Core Decompression Improves Outcome for Patients with Early-Stage Osteonecrosis of the Femoral Head: A Comparative Study. The Journal of Arthroplasty 30(9):11–15

86. Talathi NS, Kamath AF (2018) Autologous stem cell implantation with core decompression for avascular necrosis of the femoral head. J Clin Orthop Trauma 9(4):349–352

87. Tomaru Y, T Y, H S, H K, K A, H W, H A, M Y, H M (2022) Comparison Between Concentrated Autologous Bone Marrow Aspirate Transplantation as a Hip Preserving Surgery and Natural Course in Idiopathic Osteonecrosis of the Femoral Head. CureusDOI: 10.7759/cureus.24658

88. Tomaru Y, Yoshioka T, Nanakamura J, Sugaya H, Hagiwara S, Nawata K, Ohtori S, Yamazaki M, Mishima H (2021) Concentrated autologous bone marrow aspirate transplantation versus conservative treatment for corticosteroid-associated osteonecrosis of the femoral head in systemic lupus erythematosus. Journal of Rural Medicine 16(1):1–7

89. Tomaru Y, Yoshioka T, Sugaya H, Kumagai H, Hyodo K, Aoto K, Wada H, Akaogi H, Yamazaki M, Mishima H (2019) Ten-year results of concentrated autologous bone marrow aspirate transplantation for osteonecrosis of the femoral head: a retrospective study. BMC Musculoskelet Disord 20(1):410

90. Ulusoy İ, Yılmaz M, Kıvrak A (2023) Efficacy of autologous stem cell therapy in femoral head avascular necrosis: a comparative study. J Orthop Surg Res 18(1):799

91. Wang B-L, Sun W, Shi Z-C, Zhang N-F, Yue D-B, Guo W-S, Xu S-Q, Lou J-N, Li Z-R (2010) Treatment of nontraumatic osteonecrosis of the femoral head with the implantation of core decompression and concentrated autologous bone marrow containing mononuclear cells. Arch Orthop Trauma Surg 130(7):859–865

92. Wang T, Wang W, Yin ZS (2014) Treatment of osteonecrosis of the femoral head with thorough debridement, bone grafting and bone-marrow mononuclear cells implantation. Eur J Orthop Surg Traumatol 24(2):197–202

93. Wu ZY, Sun Q, Liu M, Grottkau BE, He ZX, Zou Q, Ye C (2020) Correlation between the efficacy of stem cell therapy for osteonecrosis of the femoral head and cell viability. BMC Musculoskelet Disord 21:55

94. Xian H, Luo D, Wang L, Cheng W, Zhai W, Lian K, Lin D (2020) Platelet-Rich Plasma-Incorporated Autologous Granular Bone Grafts Improve Outcomes of Post-Traumatic Osteonecrosis of the Femoral Head. The Journal of Arthroplasty 35(2):325–330

95. Yamasaki T, Yasunaga Y, Ishikawa M, Hamaki T, Ochi M (2010) Bone-marrow-derived mononuclear cells with a porous hydroxyapatite scaffold for the treatment of osteonecrosis of the femoral head: A PRELIMINARY STUDY. The Journal of Bone & Joint Surgery British Volume 92-B(3):337–341

96. Yamasaki T, Yasunaga Y, Terayama H, Ito Y, Ishikawa M, Adachi N, Ochi M (2008) Transplantation of bone marrow mononuclear cells enables simultaneous treatment with osteotomy for osteonecrosis of the bilateral femoral head. Med Sci Monit 14(4):CS23-30

97. Yan D, Chen L, Li Z, Guo W, Sun W (2015) Autologous mesenchymal stem cell implantation in the management of osteonecrosis of the femoral head. Current Orthopaedic Practice 26(3):265

98. Yang Y-L, Wang H, Hu Q, Yu D, Zhang H-L, Wang B, Wang C, Wang B, Cui J-H, Zhu C, Liu X-H (2022) Efficacy of robot-assisted core decompression combined with human umbilical cord-derived mesenchymal stem cell transplantation for osteonecrosis of the femoral head. Eur Rev Med Pharmacol Sci 26(12):4197–4206

99. Ying J, Wang P, Ding Q, Shen J, O'Keefe RJ, Chen D, Tong P, Jin H (2020) Peripheral Blood Stem Cell Therapy Does Not Improve Outcomes of Femoral Head Osteonecrosis With Cap-Shaped Separated Cartilage Defect. Journal of Orthopaedic Research 38(2):269–276

100. Yoon PW, Kang JY, Kim C-H, Lee SJ, Yoo JJ, Kim HJ, Kang SK, Min JH, Yoon KS (2021) Culture-Expanded Autologous Adipose-Derived Mesenchymal Stem Cell Treatment for Osteonecrosis of the Femoral Head. Clin Orthop Surg 13(1):37–46

101. Yoshioka T, Mishima H, Akaogi H, Sakai S, Li M, Ochiai N (2011) Concentrated autologous bone marrow aspirate transplantation treatment for corticosteroid-induced osteonecrosis of the femoral head in systemic lupus erythematosus. International Orthopaedics (SICOT) 35(6):823–829

102. Yoshizawa T, Yoshioka T, Sugaya H, Nishino T, Tomaru Y, Wada H, Akaogi H, Yamazaki M, Mishima H (2022) Total Hip Arthroplasty After Failed Hip-Preserving Surgery with Concentrated Autologous Bone Marrow Aspirate Transplantation for Osteonecrosis of the Femoral Head: A Retrospective Study. Indian J Orthop 56(7):1251–1258

103. Zhang W, Zheng C, Yu T, Zhang H, Huang J, Chen L, Tong P, Zhen G (2022) The therapeutic effect of adipose-derived lipoaspirate cells in femoral head necrosis by improving angiogenesis. Front Cell Dev Biol 10:1014789

104. Zhao D (2022) Autologous stem cells combined with core decompression on patients with osteonecrosis of the femoral head. Acta Medica MediterraneaDOI: 10.19193/0393-6384_2022_4_425

105. Zhao D, Cui D, Wang B, Tian F, Guo L, Yang L, Liu B, Yu X (2012) Treatment of early stage osteonecrosis of the femoral head with autologous implantation of bone marrow-derived and cultured mesenchymal stem cells. Bone 50(1):325–330

106. Zhao D, Liu B, Wang B, Yang L, Xie H, Huang S, Zhang Y, Wei X (2015) Autologous Bone Marrow Mesenchymal Stem Cells Associated with Tantalum Rod Implantation and Vascularized Iliac Grafting for the Treatment of End-Stage Osteonecrosis of the Femoral Head. BioMed Research International 2015:e240506
