# Supplementary material for: Orthobiologic therapies delay the need for hip arthroplasty in patients with avascular necrosis of the femoral head: A systematic review and survival analysis
Source: Knee Surg Sports Traumatol Arthrosc. 2024 Nov 14;33(3):1112–27. doi: 10.1002/ksa.12532 (PMC11848991; doi:10.1002/ksa.12532)
Supplement: Supplementary file 2 — Supporting information. [file KSA-33-1112-s002.docx]

**Table S2**. Effect size and heterogeneity of the level II survival analyses.

| **Analysis** | **Pouled**  **Hazard Ratio**  ***(effect size)*** | **Lower 95% CI limit** | **Upper 95%CI limit** | **P value** | **I^2^** |
| --- | --- | --- | --- | --- | --- |
| **Orthobiologics**  **Vs**  **Controls** | 1,90 | 1.47 | 2.44 | <0.0005 | 0,13 |
| **BMAC**  **Vs**  **Controls** | 1.99 | 1.47 | 2.70 | <0.0005 | 0.26 |
| **BM-MSC**  **Vs**  **Controls** | 1.63 | 0.59 | 4.55 | 0.349 | 0.10 |
| **PRP**  **Vs**  **Controls** | 1.22 | 0.52 | 2.84 | 0.643 | 0.00 |

BMAC, bone marrow aspirate concentrate; BM-MSCs, bone marrow-derived mesenchymal stromal cells; CI, confidence interval; I, heterogeneity index; PRP, platelet-rich plasma.
